# Specific drug delivery efficiently induced human breast tumor regression using a lipoplex by non-covalent association with anti-tumor antibodies

**DOI:** 10.1186/s12951-019-0457-3

**Published:** 2019-02-06

**Authors:** Yu-Ling Lin, Nu-Man Tsai, Chia-Hung Chen, Yen-Ku Liu, Chung-Jen Lee, Yi-Lin Chan, Yu-Shan Wang, Yuan-Ching Chang, Chi-Hsin Lin, Tse-Hung Huang, Chao Ching Wang, Kwan-Hwa Chi, Kuang-Wen Liao

**Affiliations:** 1 Agricultural Biotechnology Research Center, Academia Sinica, Taipei, 11529 Taiwan, ROC; 2 School of Medical Laboratory and Biotechnology, Chung Shan Medical University, Taichung, 40201 Taiwan, ROC; 3 Department of Pathology and Clinical Laboratory, Chung Shan Medical University Hospital, Taichung, 40201 Taiwan, ROC; 4 Institute of Molecular Medicine and Bioengineering, National Chiao Tung University, Hsinchu, 30068 Taiwan, ROC; 5 Department of Biological Science and Technology, National Chiao Tung University, No.75 Po-Ai Street, Hsinchu, 30068 Taiwan, ROC; 6 Department of Nursing, Tzu Chi College of Technology, Hualien, 97005 Taiwan, ROC; 7 Department of Life Science, Chinese Culture University, Taipei, 11114 Taiwan, ROC; 8 Department of Radiation Therapy and Oncology, Shin-Kong Wu Ho-Su Memorial Hospital, No.95, Wenchang Rd., Shilin Dist., Taipei City, 11101 Taiwan, ROC; 9 Department of Surgery, Mackay Memorial Hospital, Taipei, 10491 Taiwan, ROC; 10 Department of Medical Research, MacKay Memorial Hospital, Taipei, 10491 Taiwan, ROC; 11 Department of Traditional Chinese Medicine, Chang Gung Memorial Hospital, Keelung, 20401 Taiwan, ROC; 12 School of Traditional Chinese Medicine, Chang Gung University, Taoyuan, 33302 Taiwan, ROC; 13 School of Nursing, National Taipei University of Nursing and Health Sciences, Taipei, 11219 Taiwan, ROC; 14 Graduate Institute of Medicine, College of Medicine, Kaohsiung Medical University, Kaohsiung, 80708 Taiwan, ROC; 15 Center for Intelligent Drug Systems and Smart Bio-devices, National Chiao Tung University, Hsinchu, 30068 Taiwan, ROC

**Keywords:** Lipo-PEG-PEI complex, Curcumin, Doxorubicin, Herceptin, Drug delivery

## Abstract

**Background:**

A cationic liposome-PEG-PEI complex (LPPC) was employed as a carrier for achieving targeted delivery of drug to human epidermal growth factor receptor-2 (HER2/neu)-expressing breast cancer cells. LPPC can be easily loaded with an anti-tumor drug and non-covalently associated with an anti-tumor antibody such as Herceptin that is clinically used to rapidly form immunoparticles within 1 h.

**Results:**

Drug-loaded LPPC have an average size about 250 nm and a zeta potential of about 40 mV. Herceptin was complexed onto surface of the LPPC to form the drug/LPPC/Herceptin complexes. The size of curcumin/LPPC/Herceptin complexes were 280 nm and the zeta potentials were about 23 mV. Targeting ability of this delivery system was demonstrated through specific binding on surface of cells and IVIS images in vivo, which showed specific binding in HER2-positive SKBR3 cells as compared to HER2-negative Hs578T cells. Only the drug/LPPC/Herceptin complexes displayed dramatically increased the cytotoxic activity in cancer cells. Both in vitro and in vivo results indicated that Herceptin adsorbed on LPPC directed the immunocomplex towards HER2/neu-positive cells but not HER2/neu-negative cells. The complexes with either component (curcumin or doxorubicin) used in the LPPC-delivery system provided a better therapeutic efficacy compared to the drug treatment alone and other treatment groups, including clinical dosages of Herceptin and LipoDox, in a xenografted model.

**Conclusions:**

LPPC displays important clinical implications by easily introducing a specific targeting characteristic to drugs utilized for breast cancer therapy.

**Electronic supplementary material:**

The online version of this article (10.1186/s12951-019-0457-3) contains supplementary material, which is available to authorized users.

## Background

Most doctors consider chemotherapy as an efficient and essential strategy of clinical therapy for patients with metastatic tumors. Anti-tumor drugs are used to inhibit tumor growth, suppress angiogenesis in tumors or induce apoptosis in tumor cells spread throughout the patient’s body. There is no doubt that the relative amount of anti-tumor drugs accumulated within the tumor area is crucial for therapeutic efficacy. Although the administration of high doses of anti-tumor drugs may indeed increase the levels of cytotoxicity in cancer cells, this also increases the risk of normal organ failure. Circumventing this issue, drug-encapsulated liposomes and other nanoparticles have been found to selectively target tumors via leaky vessels, thereby enhancing the anti-tumor efficacy and reducing the side effects in normal tissues [1–4]. Furthermore, covalent conjugation of the targeting molecules, such as with cell-specific peptides (RGD and GE11 peptides) [5–7] and monoclonal antibodies (anti-HER2/neu and anti-EGFR antibodies) [1, 8–10], has further improved the efficacy compared with drug-encapsulated particles.

The available targetable nanoparticles are produced using thiol, carboxylic acid or amine groups for the covalent linkage, which may attenuate the activity of certain targeting molecules during the coupling reaction [11–13]. Additionally, prior processing of the nanoparticle and targeting molecules requires multiple steps and are therefore both time-consuming and sample-consuming [8, 14, 15]. In contrast to covalent conjugation, the non-covalent method of targeting molecules with cationic nanoparticles may circumvent this issue by electrostatic interaction if the targeting molecules stably exist on nanoparticles. However, the weak interaction between the targeting molecules and nanoparticles is usually interrupted by certain substances within the microenvironment, including salts and proteins, which cause the defluxion of targeting molecules from the liposomes and influences the specific targeting [12, 16]. Previous studies have shown that IgG sonicated with phospholipids in which 4–40% of the IgG is bound to the vesicles, but the vesicle-bound IgG only retains low activities [17, 18]. To improve the system, cationic P(MDS-co-CES) micelles were developed, which bind Herceptin or TRAIL via hydrogen bonds and/or hydrophobic interactions and encapsulates doxorubicin or paclitaxel to target cancer cells in vitro [19–21]. Until now, the in vivo efficacy of this approach has not yet been displayed.

**Fig. 1:**
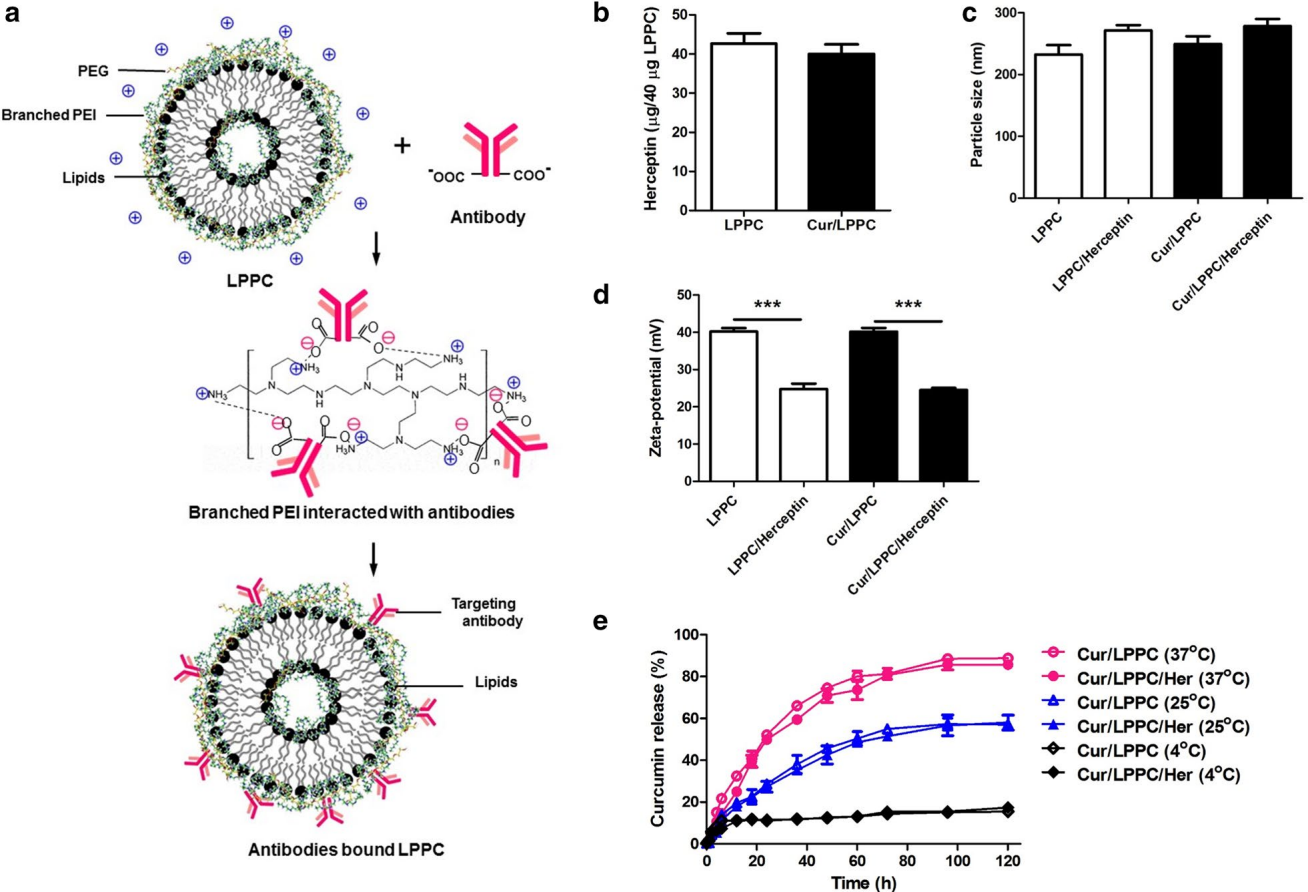
The characteristics of the drug/LPPC/targeting antibody complex. **a** The scheme for curcumin/LPPC/Herceptin preparation. PEI provides its positive charges of amine groups interact with the carboxy groups of antibodies. **b** The maximal-binding capacity of Herceptin to LPPC. LPPC (40 μg) was incubated with different amounts of Herceptin, and the maximal amount of bound protein was analyzed with the Bradford Assay. **c** The particle size and **d** zeta-potential of the drug/LPPC/targeting antibodies complex. **e** The effect of Herceptin association on curcumin release from the curcumin/LPPC complexes. Curcumin/LPPC or Curcumin/LPPC/Herceptin complexes were incubated in PBS at 4, 25 or 37 °C. As previous described in the materials and methods section, the concentration of curcumin in each supernatant was measured at various incubation time points and compared with the total curcumin concentration. All values represent the mean ± SD, ***p < 0.001 (n = 3)

In this report, we established an easy assembled immunolipoplex platform for specific cancer therapy. This lipoplex, LPPC, has been shown to strongly adsorb various biologically functional proteins on its surface and these bound proteins cannot be replaced by proteins within the environment [22]. Additionally, it has also been demonstrated that LPPC protects drug structure against oxidation and provides a strong cytotoxic effect against drug-resistant tumor cells [23, 24]. Curcumin had been reported it has anti-tumor, anti-metastasis and anti-angiogenesis activities. In addition, curcumin also had been encapsulated into various nanosystems to increase the therapeutic efficacy and overcome the drug resistance [23, 24]. We combined such characteristics to develop a specific drug delivery system, successfully fabricated by loading a model drug, curcumin, into LPPC and stably associating Herceptin onto surface of the LPPC (Fig. 1a). The branched polyethylenimine (PEI) provided positive charges from its amine groups to associate with the carboxy groups of antibodies by electrostatic interaction (Fig. 1a). Branched PEI not only provided the positive charge to associate antibodies but also made a dense reticulum with PEG to fix the protein on surface of LPPC. This drug delivery platform represents a valuable technique to rapidly prepare the anti-tumor drug-encapsulated lipoplex with targeting molecules for cancer therapy by easily loading anti-tumor drug into empty LPPC and stably adsorbing the targeting antibody. This drug/LPPC/Herceptin complex specifically target to the HER2/neu overexpressed breast cancer cells and then taken up to quickly release drug giving efficient cancer therapy.

## Experimental methods

### Chemicals and antibodies

Curcumin (≧ 94% purity), polyethylene glycol (PEG, MW 1500 and 8000), polyethyleneimine (branched PEI, MW 25,000) and 3,3′-dioctadecyloxacarbocyanine (DiO) were purchased from Sigma-Aldrich (St. Louis, MO). 1,2-Dioleoyl-*sn*-Glycero-3-Phosphocholine (DOPC) and 1,2-Dilauroyl-*sn*-Glycero-3-Phosphocholine (DLPC) were purchased from Avanti Polar Lipids (Alabaster, AL). Herceptin was purchased from Roche (Basel, Switzerland). Rabbit polyclonal antibody to human IgG F(c)-FITC was purchased from Acris Antibodies GmbH (Herford, Germany).

### Preparation of the Lipo-PEI-PEG complex (LPPC)

LPPC was prepared as previously described. Briefly, 25 mg of DOPC and DLPC were mixed in 1 ml chloroform and coated onto a round bottom flask by a rotary evaporator (EYELA, N-1000S, Tokyo, Japan) at 37 °C to yield a thin lipid film. The lipid films were hydrated by steam for 2 h and then 5 ml of PEG-PEI solution (675 mg PEI and 220 mg PEG_8000_ in deionized water) were added into the container. The lipid films were vigorously resuspended for 10 min and then the suspension was extruded through a LiposoFast extruder (Avestin Inc., Ottawa, Canada) via a 200 nm mesh 9 times. The suspensions were diluted in deionized water 50-fold and centrifuged at 5900×*g* for 5 min to remove any unincorporated substances. Finally, the pellets were resuspended with deionized water and both types of particles, curcumin/LPPC and empty LPPC, were stored at 4 °C until needed. Before use, both types of lipoplex were warmed to room temperature.

### The formation and characterization of the drug/LPPC/Herceptin complex

For drug encapsulation, 10 μl of 100 mM curcumin or 40 mg/ml Dox were mixed with 1 mg of LPPC at room temperature for 30 min. After incubation, the mixture of curcumin or Dox and LPPC were centrifuged at 5900×*g* for 5 min to remove the non-encapsulated drug. The curcumin concentration remaining in the supernatant of the solution was then measured using a spectrophotometer (Amersham Biosciences, Uppsala, Sweden) at 432 nm. The Dox concentration remaining in the supernatant of the solution was then measured using a fluorescent spectrophotometer (Hitachi, Tokyo, Japan) at Ex 470 nm/Em 590 nm. The pellets (curcumin/LPPC) were resuspended with 100 μl deionized water and stored at 4 °C.

For the adsorption of the targeting molecule, 40 μg of drug/LPPC was incubated with 200 μg of Herceptin (Roche, Basel, Switzerland) in 50 μl for 30 min. After incubation, the excess positive charges of the drug/LPPC/Herceptin complexes were reduced by PEG_1500_incubation for 30 min twice and centrifuged at 5900×*g* for 5 min to remove the excess PEG_1500_. The particle sizes and zeta potentials of the empty LPPC and curcumin/LPPC incorporated with Herceptin were determined using a Zetasizer instrument (Zetasizer 3000HS, Malvern Instruments, Malvern, UK). The measurements of 2 mg of the various LPPC complexes were taken in 200 μl deionized water at room temperature. The in vitro release of curcumin from the Curcumin/LPPC or Curcumin/LPPC/Herceptin complexes were determined as previously described [23].

### Targeting ability of LPPC/Herceptin complexes in vitro

The HER2-positive cells, MDA-MB-231, MCF7 and SKBR3, and the HER2-negative Hs578T cell lines were obtained from the Bioresource Collection and Research Center (BCRC, Hsinchu, Taiwan) and maintained according to the manufacturer’s instructions. These cell lines (3 × 10^5^ cells) were incubated with Herceptin for 30 min followed by incubation for 30 min with fluorescein-conjugated rabbit anti-human IgG (Acris Antibodies GmbH, Herford, Germany; 1: 10,000). The fluorescence was assayed via flow cytometry (Becton–Dickinson, San Jose, CA).

LPPC was first labeled with 3 mM fluorescent lipophilic dye DiO (Sigma-Aldrich, St. Louis, MO; 10 μl in 1 mg LPPC at a final volume of 110 μl) for 30 min and subsequently washed and resuspended as described above. Next, DiO-incorporated LPPC (20 μg) was complexed with either 2 μg of Herceptin or 2 μg of Rituximab (anti-human CD20 antibody) and then blocked with 20 μl of PEG_1500_ (100 mg/ml) for an additional 30 min. Various human breast tumor cells (3 × 10^5^ cells) were incubated with 20 μg of DiO/LPPC/Herceptin or DiO/LPPC/Rituximab at 4 °C for 30 min in the dark. After the cells were washed and resuspended in 1 ml DMEM, the cells were analyzed by a flow cytometry.

### Intracellular accumulation of curcumin

MCF7 cells were seeded onto glass coverslips (Nunc, USA) at a density of 2 × 10^5^ cells per disc overnight. The cells were treated with 2 ml of medium containing either curcumin, curcumin/LPPC/Rituximab or curcumin/LPPC/Herceptin at a final curcumin concentration of 2 μM. After incubation at 37 °C for 0.5, 1 or 2 h, the media was removed and the cells were washed with PBS, fixed with 4 w/w % paraformaldehyde in PBS, and imaged with a 400× magnification using a Zeiss LSM 510 META confocal microscope (Carl Zeiss, Thornwood, NY, USA).

### Specific targeting ability of LPPC/Herceptin complexes in vivo

Athymic nude mice were inoculated with 2 × 10^7^ Hs578T cells or SKBR3 cells via injection into the left and right flank of the back. Before treatment, 1 mg of LPPC was incorporated with 3 mM fluorescent lipophilic dye DiI as described above. DiI-incorporated LPPCs (100 μg) were complexed with 10 μg of antibodies for 30 min, blocked with PEG_1500_ for 30 min, and intravenously injected into the nude mice bearing tumors. Whole body imaging of the tumor-bearing mice was performed at various time points with an IVIS Spectrum System (Caliper Life Sciences Inc., Hopkinton, MA, USA). Finally, the mice were sacrificed, and the organs were collected and analyzed with an IVIS Spectrum System.

### Cytotoxicity of curcumin/LPPC

The different cell lines were seeded onto 96-well tissue culture plates at a concentration of 1 × 10^4^ cells/100 μl/well overnight. Subsequently, the cells were treated with serial concentrations of the various agents, empty LPPC, LPPC/Herceptin, curcumin/LPPC, curcumin/LPPC/Herceptin and non-encapsulated curcumin. On the other set of studies, the cells were treated with serial concentrations of the agents, like as Dox/LPPC, Dox/LPPC/Herceptin and non-encapsulated Dox. After 48 h of incubation, the levels of cell viability for each cell line was determined by MTT colorimetric assay. The cell viability values were plotted as a percentage of the untreated control.

### Animals

Female BALB/c, BALB/c/nu and NOD-SCID mice were purchased from the National Laboratory Animal Center (Taipei, Taiwan) and maintained on a 12:12-h light: dark cycle in an animal environmental control chamber (Micro-VENT IVC Systems, Allentown, NJ). Humane animal care was ensured by use of the institutional guidelines of National Chiao Tung University (NCTU). All animal studies were approved by the Institutional Animal Care and Use Committee in National Chiao Tung University (NCTU-IACUC-104034).

### Antitumor activity of curcumin/LPPC in vivo

NOD-SCID mice were inoculated with 1 × 10^7^ SKBR-3 cells via injection into the right flank. Once a tumor mass was established, the mice were intravenously injected with either PBS, Cur/LPPC/Rituximab (40 mg/kg curcumin and 4 mg/kg Rituximab), Cur/LPPC/Herceptin (40 mg/kg curcumin and 4 mg/kg Herceptin), high dose of Cur/LPPC/Herceptin (Cur-H/LPPC/Herceptin, 200 mg/kg curcumin and 4 mg/kg Herceptin) or Herceptin (9 mg/kg Herceptin, a clinical dose) once every 3 days. The tumor volumes were measured using a caliper every 2 days, and the tumor volume was calculated using the following formula: volume (mm^3^) = length × width × high. The survival were also measured every 2 days.

The tumor-bearing mice were treated with either PBS, Dox/LPPC/Herceptin (5 mg/kg Dox and 4 mg/kg Herceptin), high dose of Dox/LPPC/Herceptin (Dox-H/LPPC/Herceptin, 50 mg/kg Dox and 4 mg/kg Herceptin), Dox plus Herceptin (5 mg/kg Dox and 4 mg/kg Rituximab) or LipoDox plus Herceptin once every week as described above. Both the tumor growth and survival were assessed as described above.

### Statistical analysis

The results were analyzed using the SAS statistical software package (SAS Institute Inc., Cary, USA). The results were expressed as the mean ± SD. A *t* test was used when comparing two independent samples, and the ANOVA test was used when comparing multiple samples. Differences in which *p *< 0.05 were considered statistically significant.

## Results

### Characteristics of drug loaded LPPC/Herceptin

Anti-tumor drugs such as curcumin are easily loaded into LPPC by incubation at room temperature and remove the non-encapsulated curcumin by centrifugation. The maximal drug encapsulated capacity of 1 mg LPPC was approximately 321.6 μg curcumin (loading efficiency is 87.2%). The proteins carry capacity of Curcumin/LPPC reach 40 μg Herceptin as well as LPPC (Fig. 1b). The LPPC bound Herceptin was stable on the LPPC or Curcumin/LPPC (Additional file 1: Figure S1). Compared to the empty LPPC, the particle sizes of Cur/LPPC/Herceptin were slightly increased (Fig. 1c and Additional file 1: Table S1), but the charges of Cur/LPPC/Herceptin were dramatically decreased (Fig. 1d, and Additional file 1: Table S1). Figure 1e and Additional file 1: Figure S2 showed the encapsulated drug slowly released from LPPC and the adsorption of Herceptin did not affect drug release from LPPC.

### Targeting ability of LPPC-based complexes in vitro and in vivo

**Fig. 2:**
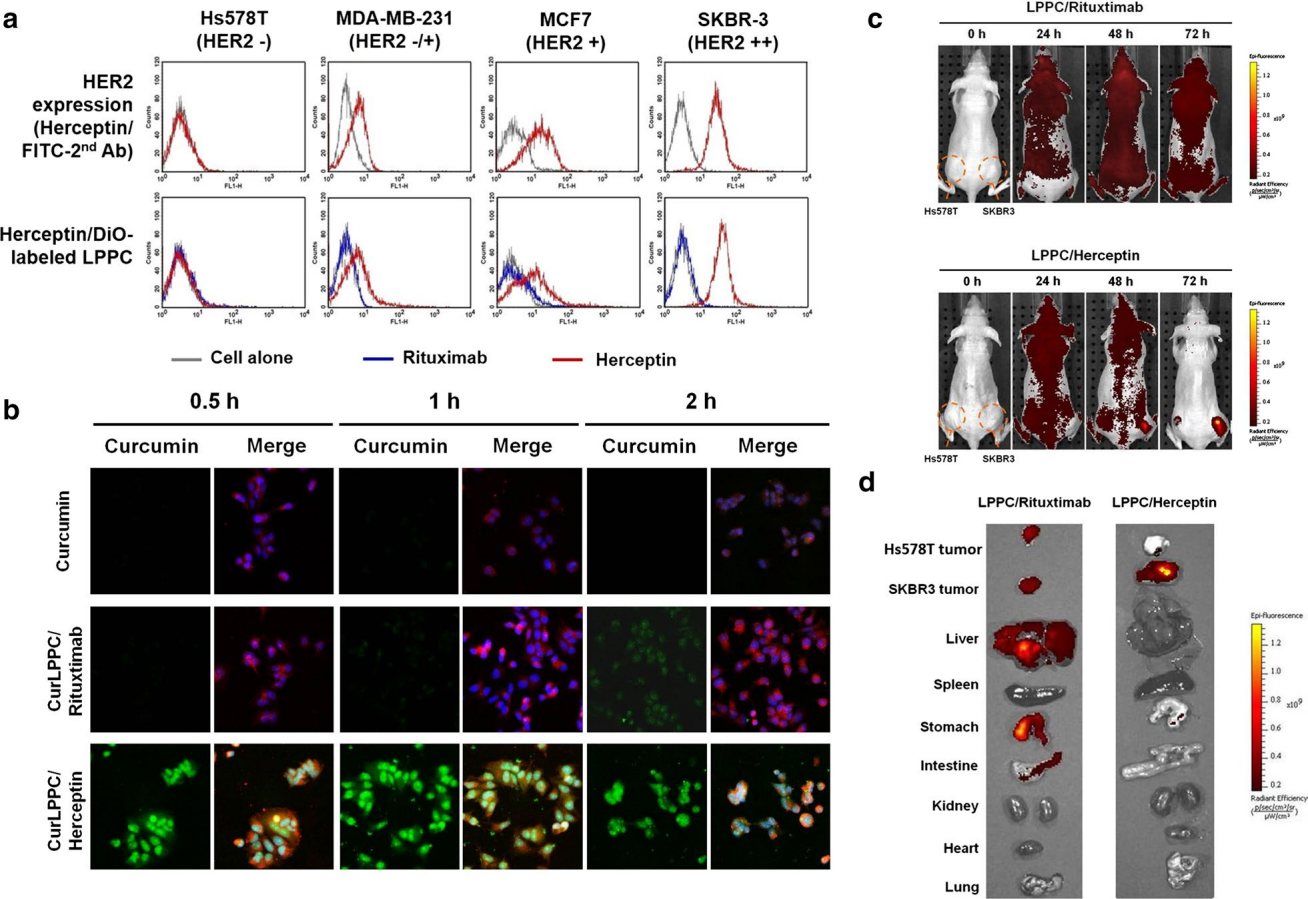
Targeting ability of the drug-loaded immunolipoplex. **a** The effect of antibody association with immunocomplexes on cell targeting in vitro. The HER2/neu receptor expressed on different cancer cell lines were indirectly probed with the humanized antibodies, Herceptin or Rituximab, and FITC-conjugated goat anti-human IgG antibody. Herceptin or Rituximab were adsorbed on DiO-labeling LPPC to monitor their ability to target breast cancer cell lines when associated with LPPC complexes. **b** The intracellular accumulation of curcumin. MCF-7 cells were treated with curcumin, curcumin/LPPC/Rituximab or curcumin/LPPC/Herceptin at equal concentrations of curcumin. The cell membranes were stained with red fluorescent dye DiI, the nuclei were stained with DAPI, and the cellular distribution of curcumin is shown as green fluorescence signal. The cells were imaged using a confocal microscope. **c** Targeting ability of LPPC in vivo. DiI-labeled LPPC/Rituximab or DiI-labeled LPPC/Herceptin complexes were i.v. injected into athymic nude mice bearing HER2-negative Hs578T cell and HER2-positive SKBR3 cell-induced tumors. The images were obtained by IVIS at 0, 24, 48 and 72 h after injection. The photon counts of each mouse are indicated by the pseudo-color scales. **d** After 72 h, the organs and tumors isolated from the treated nude mice were imaged by IVIS

To determine the efficacy of the LPPC-based complexes for drug targeting in vitro, the fluorescence dye DiO labeled-LPPC/Herceptin complexes were first determined their abilities to interact with the various breast cancer cells with different surface expression levels of HER2/neu (Fig. 2a, above panel). The DiO-LPPC/Herceptin complexes only reacted with the HER2-positive cells and displayed the florescent intensities as high as the antigen density on cells surface (Fig. 2a, below panel). In contrast, LPPC complexed with the negative antibody, Rituximab (Anti-CD20), failed to target both the HER2-positive and HER2-negative cells, which indicated that the DiO-LPPC/Herceptin complex specifically targeted HER2-positive cells in vitro. Furthermore, loading curcumin into the LPPC did not interfere with the specific targeting of the immunocomplex (Additional file 1: Figure S3). As assessed by confocal microscope, Herceptin association not only specifically directed the curcumin/LPPC complex to bind HER2-positive cells but also dramatically facilitated curcumin accumulation in the cytosol compared with Rituximab (Fig. 2b).

To examine the specific targeting ability of the LPPC/Herceptin complex in vivo, DiI-labeled LPPC/Herceptin or LPPC/Rituximab complexes were used to probe HER2-positive tumors. The results in Fig. 2c show that only Herceptin but not Rituximab directed the DiI-labeled LPPC complexes to the SKBR3 tumors (HER2-positive) in 72 h, but both failed to result in significant fluorescence in Hs578T tumors (HER2-negative). Later, their organs or tumors were collected to further analysis. The results showed that Rituximab-based LPPC complexes caused the non-specific accumulations in organs including liver, stomach, intestine, and both tumors. In contrast to Rituximab, Herceptin resulted in the specific accumulation in HER2-positive SKBR3 tumors but not normal organs (Fig. 2d). This platform does not involve covalent linkers for any component or step, including the preparation of the LPPC, drug encapsulation, and targeting antibody association. The results of Fig. 2a and Additional file 1: Figure S4 show the target molecules associated on LPPC can be changed easily and such immunocomplexes are stable enough to target antigen-positive tumors in vivo.

### The cytotoxic activity of LPPC-based complex in tumor cells in vitro

**Fig. 3:**
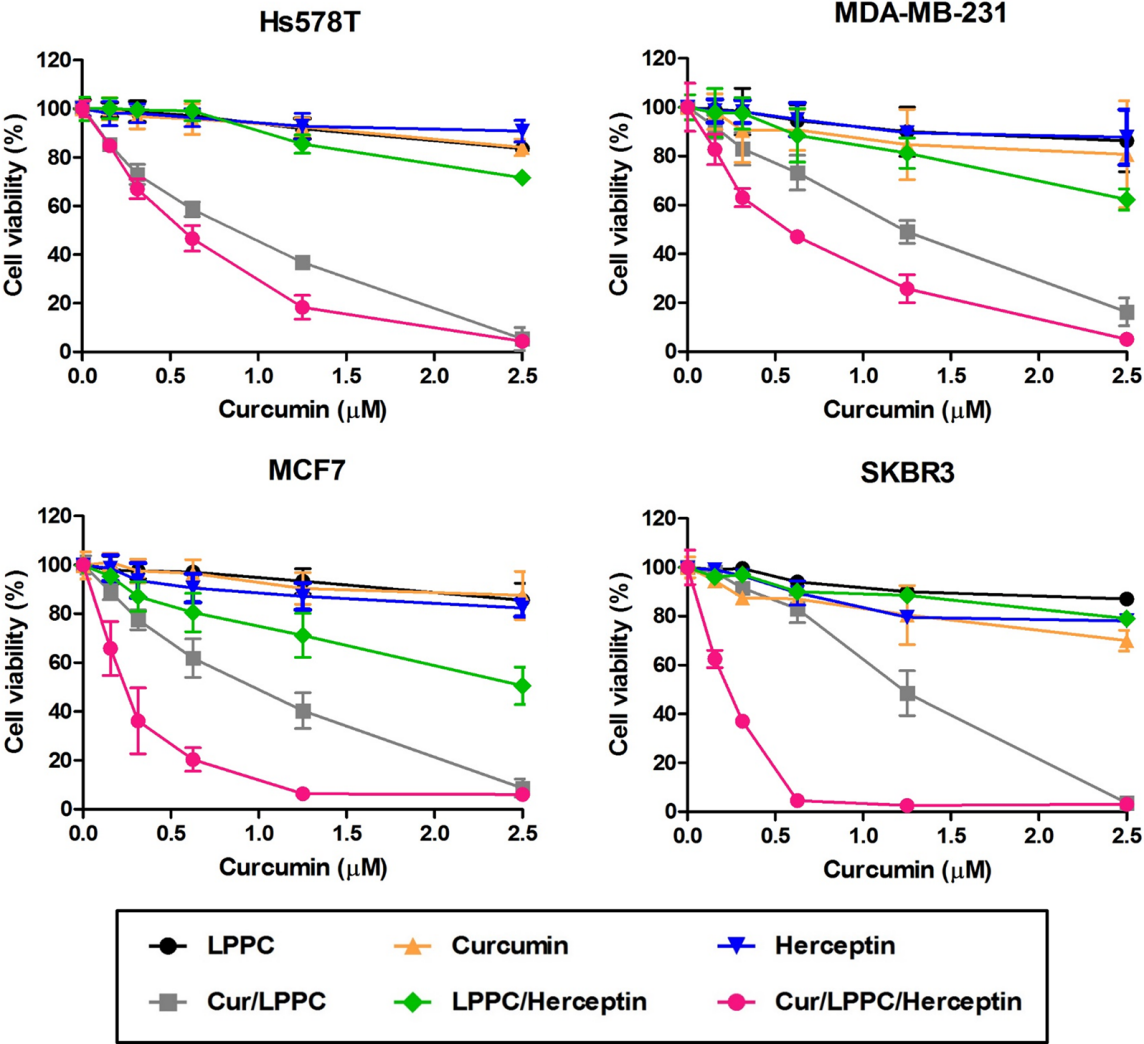
Cytotoxic effects of Cur/LPPC/Herceptin on HER2-negative or HER2-positive cell lines. Hs578T (HER2−), MDA-MB-231 (HER2 −/+), MCF7 (HER2+), and SKBR-3 (HER2++) cells were treated with 0 to 2.5 μM curcumin for the treatment of Cur/LPPC/Herceptin for 48 h. The dosages of LPPC, Herceptin, and LPPC/Herceptin were the same as those used for the Cur/LPPC/Herceptin treatments. Cell viability was assessed by MTT assay. All values represent the mean ± SD (n = 6)

To value the cytotoxic effects of Curcumin/LPPC/Herceptin complexes, different breast cancer cells with various HER2 expressions were determined their responses to the immunocomplex. The curcumin/LPPC complexes with Herceptin dramatically enhanced the cytotoxic effects of LPPC-encapsulated curcumin on HER2-positive cells (Fig. 3, Additional file 1: Table S2). Additionally, doxorubicin (Dox) was also encapsulated in LPPC to compose Dox/LPPC complexes. Dox/LPPC complexes containing approximately 205 μg Dox (per 1 mg LPPC, loading efficiency is 51.2%) and their Herceptin carry capacities, particle sizes, zeta-potential changes, PDI and drug release profiles were similar to Cur/LPPC (Additional file 1: Figure S5 and Table S1). Dox/LPPC/Herceptin complexes were performed the better cytotoxic activity against HER2-positive breast cancer cells (Additional file 1: Figure S6 and Table S3). Both results of Fig. 3 and Additional file 1: Figure S6 showed the proportion of drug/LPPC/Herceptin complexes-killed cells corresponded to the density of HER2-positive cells.

### The suppression of tumor growth using a LPPC-based complex in vivo

**Fig. 4:**
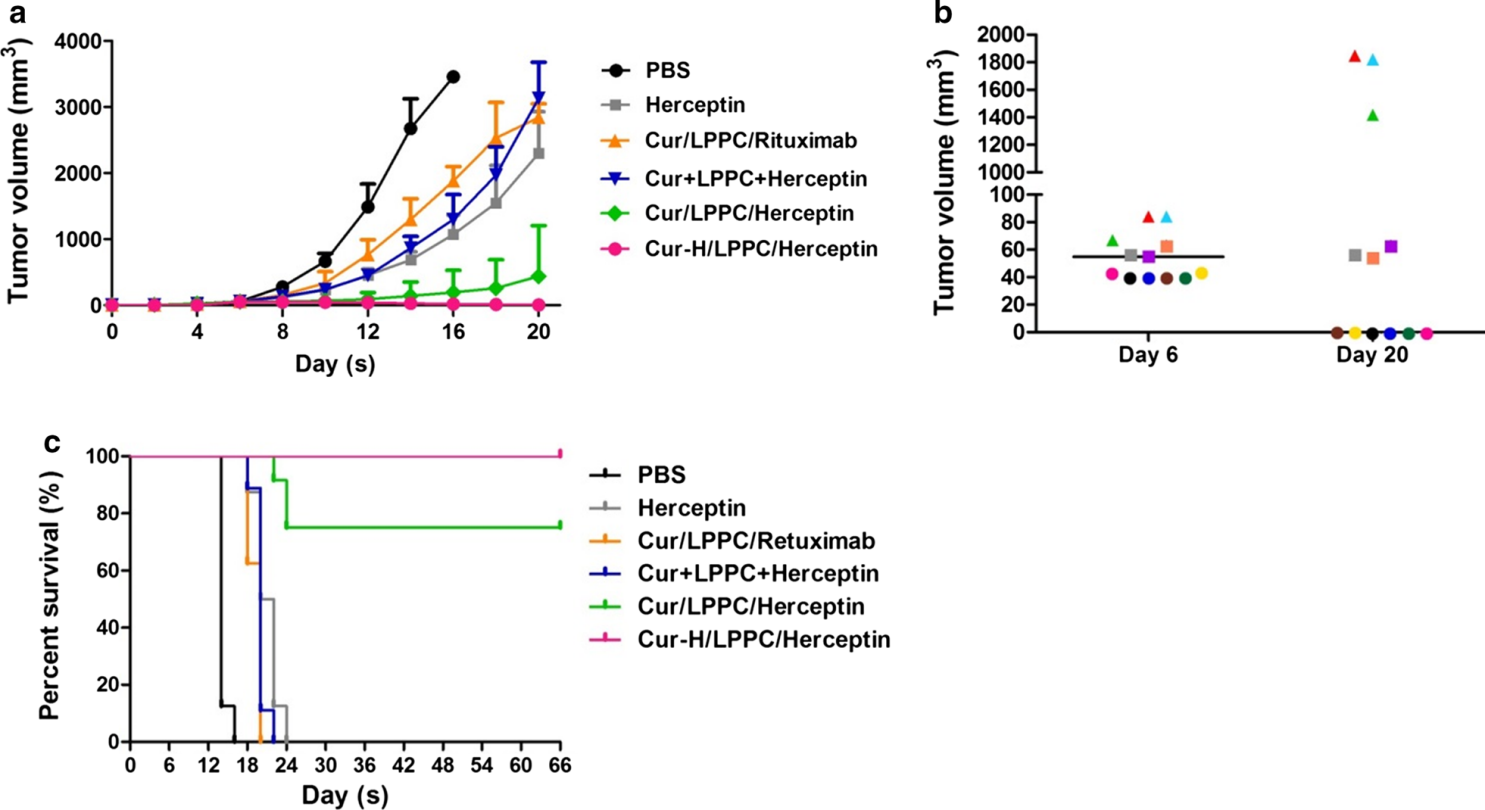
The effects of curcumin-loaded immunolipoplex on tumor growth in vivo. **a** The inhibition of curcumin/LPPC/Herceptin on tumor growth. NOD-SCID mice bearing SKBR-3 tumors (the average tumor size was 55 mm^3^ for each group) were treated on day 6 with either Cur/LPPC/Rituximab (40 mg/kg curcumin and 4 mg/kg Rituximab), Cur/LPPC/Herceptin (40 mg/kg curcumin and 4 mg/kg Herceptin), Cur + LPPC + Herceptin (40 mg/kg curcumin and 4 mg/kg Herceptin), Cur-H/LPPC/Herceptin (200 mg/kg curcumin and 4 mg/kg Herceptin), or 9 mg/kg Herceptin (clinical dose of Herceptin) via i.v. injection once every 3 ays. The tumor volumes were measured every 2 days after treatment. All values represent the mean ± SD (n = 8). **b** The tumor sizes observed in the mice in the curcumin/LPPC/Herceptin treatment group on day 6 and day 20 are shown. Each color represents an individual treated mouse, and the line indicates the average tumor size (n = 12). **c** Mouse survival was monitored daily. The mice were sacrificed when the tumors reached over 2500 mm^3^ in size

The xenograft model of the SKBR-3 human breast cancer cell line was used to evaluate the therapeutic effect of the LPPC-based complex in vivo. Figure 4a showed that Herceptin at a clinical dose (9 mg/kg) and Curcumin/LPPC with control antibody (Rituximab) leaded to 75% and 55% tumor growth inhibition compared with PBS at 16 days. Treatment with curcumin/LPPC/Herceptin (containing 40 mg/kg curcumin, green line) significantly inhibited tumor growth to result in 50% of treated mice were tumor free during 66 days which is 4 times lifespan of tumor-bearing mice with PBS treatments, and 94% inhibition of tumor growth for other 50% of curcumin/LPPC/Herceptin treated mice compared with PBS at 16 days. Interestingly, the therapeutic efficacy of curcumin/LPPC/Herceptin was dependent on tumor size-dependent (Fig. 4b). After treatment, the mouse with tumor smaller than 60 mm^3^ in the beginning of treatment was tumor free. Curcumin/LPPC/Herceptin stopped tumor growing while the start size was approximately 60 mm^3^. Curcumin/LPPC/Herceptin significantly slowed the tumor growth even the start size of tumor was larger than 60 mm^3^. The animal treated with high dose of curcumin/LPPC/Herceptin (200 mg/kg curcumin), 100% of tumor-free animals observed. The eliminated tumors in 40 mg/kg or 200 mg/kg curcumin/LPPC/Herceptin groups did not recurrence during 66 days. Moreover, treatment with Curcumin/LPPC/Herceptin significantly extended the lifespan of tumor-bearing mice to more than 3.5-fold that of the mice in the PBS group (Fig. 4c).

**Fig. 5:**
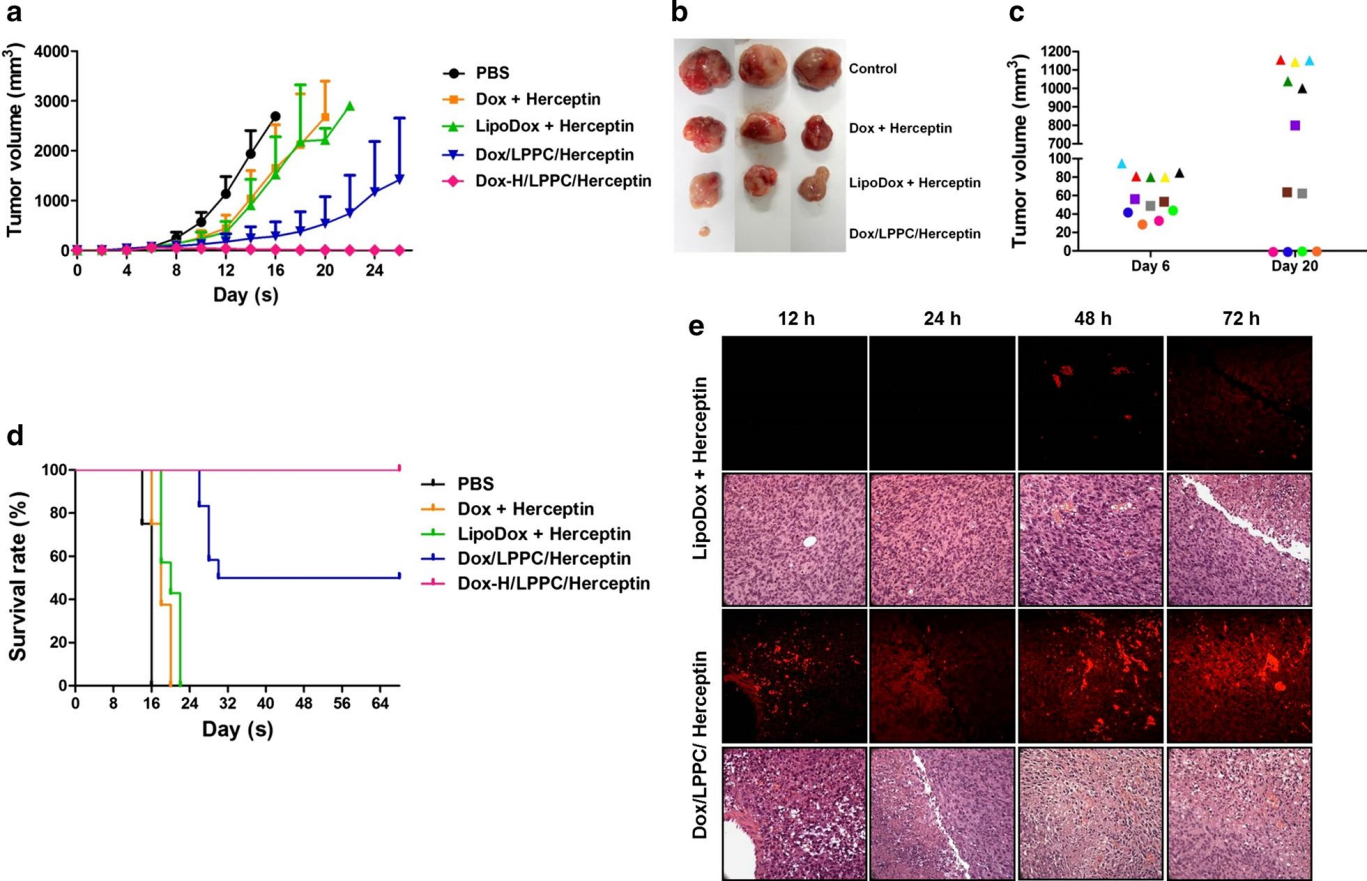
The effects of doxorubicin-loaded immunolipoplex on tumor growth in vivo. **a** The inhibition of Dox/LPPC/Herceptin on tumor growth. NOD-SCID mice bearing SKBR-3 cells were treated on day 6 with either Dox/LPPC/Rituximab (5 mg/kg Dox and 4 mg/kg Rituximab), Dox/LPPC/Herceptin (5 mg/kg Dox and 4 mg/kg Herceptin), Dox-H/LPPC/Herceptin (50 mg/kg Dox and 4 mg/kg Herceptin) or 5 mg/kg LipoDox by i.v. injection once a week. The tumor volumes were measured every 2 days after treatment. All values represent the mean ± SD (n = 8). **b** The tumors of the mice with different treatments were removed and are shown at day 16. **c** The tumor sizes of the mice after treatment with Dox/LPPC/Herceptin are shown at days 6 and 20. Each color represents an individual treated mouse (n = 12). **d** Mouse survival was monitored daily. The mice were sacrificed when the tumor sizes exceeded 2500 mm^3^. **e** Mice bearing SKBR-3 cells were treated with LipoDox plus either Herceptin or Dox/LPPC/Herceptin. The mice were scarified at different time points after treatment and the pathologic changes in tumors were examined by H&E staining and observed using a light microscope (×400). The distribution of doxorubicin within the tumors was imaged using a fluorescent light microscope (×400)

Doxorubicin, a chemotherapeutic drug used to treat human breast cancer, was encapsulated into LPPC as described above. After treatment, the Doxorubicin/LPPC/Herceptin immunocomplexes significantly suppressed the SKBR-3 tumor growth (Fig. 5a, b); 25% of animals were tumor-free, and 25% of animals displayed an arrest in tumor growth (Fig. 5c). Other treatments were used to compare the therapeutic efficacy of the LPPC-based therapy, and the results indicated that the clinical dosage for Dox or LipoDox reduced the rate of tumor growth but did not yield a complete disappearance of the tumor or arrest tumor growth (Fig. 5a). Doxorubicin/LPPC/Herceptin treatment also extended the lifespan of mice bearing SKBR-3 tumors to 3.5-fold compared with the mice in the PBS treatment group (Fig. 5d). Pathology results showed that the doxorubicin distribution in the tumor area correlated with the vessel position (Fig. 5e), while the distribution of doxorubicin in healthy organs did not correlate with vessel position for both treatments (Additional file 1: Figure S7). In contrast, the treatments with Dox/LPPC/Herceptin induced increased drug accumulation rapidly in the tumor area via vessel transport compared with LipoDox plus Herceptin (Fig. 5e). Therefore, the treatments with Dox/LPPC/Herceptin induced increased damage to vessels, thereby causing both RBC spillage and reducing the vessel numbers within the tumor (Additional file 1: Figure S8), which may also promote enhanced tumor regression. Additionally, the accumulation of high levels of doxorubicin in tumors via Dox/LPPC/Herceptin treatment induced severe levels of tumor cell death via both apoptosis and necrosis (Additional file 1: Figure S9). Such tumor cells should not be able to successfully resist drug attack, which may be another reason for the increased tumor death following Dox/LPPC/Herceptin treatment.

### Biosafety of LPPC/Herceptin for targeting therapy

Although cationic liposome-based drug delivery particles have been revealed that they provided the better efficacy of drug delivery and therapy, cationic nanoparticles will non-specifically interact with the molecules on cell membranes of blood cells to result in agglutination, which may be dangerous to threat the life of the treated animal. The cationic LPPC particles were firstly examined their safety in this view. The results in Additional file 1: Figures S10 and S11 indicated even high dose of LPPC-PEG_1500_ (5 mg LPPC per mouse) did not change the body weight and cause organ damages as well as PBS treatment. Meanwhile, the results also showed ten biochemical indexes such as albumin, total protein, glucose, alanine aminotransferase, aspartate aminotransferase, creatine kinase, lactate dehydrogenase, CRE, urea and lactate were in the normal ranges for the LPPC-PEG_1500_ treated mice (Additional file 1: Tables S4–S6**)**. Maximal tolerance of LPPC-PEG_1500_ in mice is over 30 mg per mouse for the acute toxicity. These results pointed out that PEG_1500_ addition significantly reduce the cytotoxic effects of LPPC and achieve the future application for drug delivery.

### Pharmacokinetics of Cur/LPPC/Herceptin

Sequentially, pharmacokinetics of drug/immunocomplex was identified, and Additional file 1: Table S7 showed the half-life of curcumin in sera from the curcumin/LPPC/Herceptin was about 14 h, it is more than the half-life of non-encapsulated curcumin (less than 1 h) and similar to curcumin/LPPC. All data showed that LPPC encapsulation reduces the clearance of curcumin from the circulation and extend the duration of drug in the circulation, which may provide a better therapeutic efficacy in vivo compared with non-encapsulated curcumin.

## Discussion

This drug delivery platform represents a valuable technique to rapidly prepare the anti-tumor drug-encapsulated lipoplex with targeting molecules for cancer therapy by easily loading anti-tumor drug into empty LPPC and adsorbing the targeting antibody. The unloaded drugs and unbound targeting molecules were easily removed by centrifugation. This platform does not involve covalent linkers for any component or step, including the preparation of the LPPC, drug encapsulation, and targeting antibody association. However, the results show that such immunocomplexes are stable enough to target antigen-positive tumors in vivo. Figures 2, 4 and 5 clearly show that the LPPC-based drug delivery system with the adsorption of targeting molecules not only enhances the specific targeting but also displays efficient therapeutic efficacy in vivo.

Moreover, even without adsorbing targeting antibodies, LPPC encapsulation has been shown to suppress tumor growth more effectively than non-encapsulated drug by introducing higher concentrations of the drug into the tumor area. This study also showed that the adsorption of Herceptin on LPPC dramatically improves the cytotoxic effects of the drug on tumor cells compared to no adsorption of the targeting antibody (Fig. 3) by enhancing drug translocation into the tumor area (Fig. 2b). Thus, both encapsulation and the delivery of anti-tumor drug seem to yield increased therapeutic efficacy than free diffusion. Some clinically applied anti-tumor drugs fail to exert activity in vivo. If they can be modified via encapsulation or association with targeting molecules, they can provide increased therapeutic benefits for patients. The LPPC platform represents an available technique to examine the efficacy of encapsulation or targeting for clinically applied drugs in an experimental animal model.

LPPC associated with the targeting molecule Herceptin on its surface by adsorption, which is important to increase the cytotoxic effects on breast cancer cells. The key component of the adsorbing capability of LPPC is the PEI component. Without PEI incorporation into LPPC, LPPC loses the capacity to bind proteins on its surface [22]. Therefore, the PEI component of LPPC not only plays a role in protein adsorption but also facilitates the penetration of the encapsulated drug across the cell membrane [23]. However, PEI is highly cytotoxic due to the excess positive charges of PEI, which may be dangerous to threat the life of the treated animal. In order to reach the goal of the future application for specific drug delivery, the tissue damage of LPPC treatment must be evaluated. After treatment with LPPC mixed with PEG_1500_, the mice were weighted and their blood and organs were collected to evaluate the toxicity.

Unlike covenant conjugation, LPPC associated with the targeting molecule Herceptin on its surface by adsorption, which is important to increase the cytotoxic effects on breast cancer cells. In a previous publication, we showed that the key component of the adsorbing capability of LPPC is the PEI component. Without PEI incorporation into LPPC, LPPC loses the capacity to bind proteins on its surface. Compared with the liposome without PEI-coating, the results have further indicated that the PEI component of LPPC not only plays a role in protein adsorption but also facilitates the penetration of the encapsulated drug across the cell membrane. However, PEI is highly cytotoxic. This is due to the excess positive charges of PEI, which can be circumvented using PEG and blocking [25, 26]; moreover, PEG_1500_ blocking significantly reduces the non-specific binding and cytotoxic effects of LPPC. The pathology results show that treatments with empty LPPC did not induce organ damage in mice compared to the PBS treatments (Additional file 1: Figure S11).

Via the replacement of Herceptin with these targeting molecules, Curcumin/LPPC complexes display great potential as an improved drug delivery system for various types of cancer therapy. The increasing anti-proliferative effect of the curcumin/LPPC/Herceptin complexes may result from several possibilities: (1) LPPC-encapsulation prolongs the half-life of anti-tumor drugs (Additional file 1: Table S7), (2) Herceptin association with LPPC directs a majority of the drug-loaded LPPC to the tumor area but not healthy organs (Figs. 2c, d and 5e), and (3) the ability of the LPPC/Herceptin complexes to facilitate either drug penetration into cells or endocytosis of the drug (Fig. 2b).

Pathology results showed that the treatments with Dox/LPPC/Herceptin induced increased drug accumulation rapidly in the tumor area via vessel transport compared with LipoDox plus Herceptin. Therefore, the treatments with Dox/LPPC/Herceptin induced increased damage to vessels, thereby causing both RBC spillage and reducing the vessel numbers within the tumor (Additional file 1: Figures S7 and S8) compared with LipoDox, which may also promote enhanced tumor regression. Additionally, the accumulation of high levels of doxorubicin in tumors via Dox/LPPC/Herceptin treatment induced severe levels of tumor cell death via both apoptosis and necrosis (Additional file 1: Figure S9). Such tumor cells should not be able to successfully resist drug attack, which may be another reason for the increased tumor death following Dox/LPPC/Herceptin treatment.

With this drug delivery system, it is not only easy to encapsulate the drug and strongly adsorb targeting molecules, but it also provides a specific targeting and efficient therapeutic effect. To the best of our knowledge, no non-covalent immunoparticle has been shown to induce efficient targeting therapy in vivo as was demonstrated with the LPPC-based immunocomplex. This study demonstrates that LPPC is both an effective and available platform for the experimental investigation of specific targeting with current cancer therapies, and it is an excellent candidate for the development of targeting anti-cancer drugs in the future.

## Additional file

**Additional file 1.** Additional figures and tables.

## References

[1] Liu KC, Arivajiagane A, Wu SJ, Tzou SC, Chen CY, Wang YM (2019). Development of a novel thermal-sensitive multifunctional liposome with antibody conjugation to target EGFR-expressing tumors. Nanomed Nanotechnol Biol Med.

[2] Zabielska-Koczywas K, Lechowski R (2017). The use of liposomes and nanoparticles as drug delivery systems to improve cancer treatment in dogs and cats. Molecules (Basel, Switzerland).

[3] Sanna V, Sechi M (2012). Nanoparticle therapeutics for prostate cancer treatment. Nanomed Nanotechnol Biol Med.

[4] Li L, Ahmed B, Mehta K, Kurzrock R (2007). Liposomal curcumin with and without oxaliplatin: effects on cell growth, apoptosis, and angiogenesis in colorectal cancer. Mol Cancer Ther.

[5] Zhao H, Wang JC, Sun QS, Luo CL, Zhang Q (2009). RGD-based strategies for improving antitumor activity of paclitaxel-loaded liposomes in nude mice xenografted with human ovarian cancer. J Drug Target.

[6] Yang X, Zhang C, Li A, Wang J, Cai X (2019). Red fluorescent ZnO nanoparticle grafted with polyglycerol and conjugated RGD peptide as drug delivery vehicles for efficient target cancer therapy. Mater Sci Eng Mater Biol Appl.

[7] Biscaglia F, Rajendran S, Conflitti P, Benna C, Sommaggio R, Litti L, Mocellin S, Bocchinfuso G, Rosato A, Palleschi A (2017). Enhanced EGFR targeting activity of plasmonic nanostructures with engineered GE11 peptide. Adv Healthcare Mater.

[8] Arya G, Vandana M, Acharya S, Sahoo SK (2011). Enhanced antiproliferative activity of Herceptin (HER2)-conjugated gemcitabine-loaded chitosan nanoparticle in pancreatic cancer therapy. Nanomedicine.

[9] Li J, Xu W, Yuan X, Chen H, Song H, Wang B, Han J (2017). Polymer-lipid hybrid anti-HER2 nanoparticles for targeted salinomycin delivery to HER2-positive breast cancer stem cells and cancer cells. Int J Nanomed.

[10] Lee YH, Ma YT (2017). Synthesis, characterization, and biological verification of anti-HER2 indocyanine green-doxorubicin-loaded polyethyleneimine-coated perfluorocarbon double nanoemulsions for targeted photochemotherapy of breast cancer cells. J Nanobiotechnol.

[11] Kocbek P, Obermajer N, Cegnar M, Kos J, Kristl J (2007). Targeting cancer cells using PLGA nanoparticles surface modified with monoclonal antibody. J Control Release.

[12] Nobs L, Buchegger F, Gurny R, Allemann E (2004). Current methods for attaching targeting ligands to liposomes and nanoparticles. J Pharm Sci.

[13] Zeng J, Wang X, Wang S (2007). Self-assembled ternary complexes of plasmid DNA, low molecular weight polyethylenimine and targeting peptide for nonviral gene delivery into neurons. Biomaterials.

[14] Bae KH, Lee K, Kim C, Park TG (2011). Surface functionalized hollow manganese oxide nanoparticles for cancer targeted siRNA delivery and magnetic resonance imaging. Biomaterials.

[15] Occhipinti E, Verderio P, Natalello A, Galbiati E, Colombo M, Mazzucchelli S, Salvade A, Tortora P, Doglia SM, Prosperi D (2011). Investigating the structural biofunctionality of antibodies conjugation to magnetic nanoparticles. Nanoscale.

[16] Sun B, Ranganathan B, Feng S (2008). Multifunctional poly(d, l-lactide-*co*-glycolide)/montmorillonite (PLGA/MMT) nanoparticles decorated by Trastuzumab for targeted chemotherapy of breast cancer. Biomaterials.

[17] Huang L, Kennel SJ (1979). Binding of immunoglobulin G to phospholipid vesicles by sonication. Biochemistry.

[18] Anhorn MG, Wagner S, Kreuter J, Langer K, von Briesen H (2008). Specific targeting of HER2 overexpressing breast cancer cells with doxorubicin-loaded Trastuzumab-modified human serum albumin nanoparticles. Bioconjugate Chem.

[19] Lee AL, Wang Y, Cheng HY, Pervaiz S, Yang YY (2009). The co-delivery of paclitaxel and Herceptin using cationic micellar nanoparticles. Biomaterials.

[20] Lee AL, Wang Y, Pervaiz S, Fan W, Yang YY (2011). Synergistic anticancer effects achieved by co-delivery of TRAIL and paclitaxel using cationic polymeric micelles. Macromol Biosci.

[21] Lee AL, Dhillon SHK, Wang Y, Pervaiz S, Fan W, Yang YY (2011). Synergistic anti-cancer effects via co-delivery of TNF-related apoptosis-inducing ligand (TRAIL/Apo2L) and doxorubicin using micellar nanoparticlesw. Mol BioSyst.

[22] Liu YK, Lin YL, Chen CH, Lin CM, Ma KL, Chou FH, Tsai JS, Lin HY, Chen FR, Cheng TL (2011). A unique and potent protein binding nature of liposome 1 containing polyethylenimine and polyethylene glycol: a nondisplaceable property. Biotechnol Bioeng.

[23] Lin YL, Liu YK, Tsai NM, Hsieh JH, Chen CH, Lin CM, Liao KW (2012). A lipo-PEG-PEI complex for encapsulating curcumin that enhances its antitumor effects on curcumin-sensitive and curcumin-resistance cells. Nanomed Nanotecnol Biol Med.

[24] Lin YL, Chang KF, Huang XF, Hung CL, Chen SC, Chao WR, Liao KW, Tsai NM (2015). Liposomal *n*-butylidenephthalide protects the drug from oxidation and enhances its antitumor effects in glioblastoma multiforme. Int J Nanomed.

[25] Charles PT, Stubbs VR, Soto CM, Martin BD, White BJ, Taitt CR (2009). Reduction of non-specific protein adsorption using poly(ethylene) glycol (PEG) modified polyacrylate hydrogels in immunoassays for Staphylococcal enterotoxin B detection. Sensors (Basel, Switzerland).

[26] Cerruti M, Fissolo S, Carraro C, Ricciardi C, Majumdar A, Maboudian R (2008). Poly(ethylene glycol) monolayer formation and stability on gold and silicon nitride substrates. Langmuir.

