# Supplementary material for: Specific drug delivery efficiently induced human breast tumor regression using a lipoplex by non-covalent association with anti-tumor antibodies
Source: J Nanobiotechnology. 2019 Feb 6;17:25. doi: 10.1186/s12951-019-0457-3 (PMC6364477; doi:10.1186/s12951-019-0457-3)
Supplement: Supplementary file 1 — Additional file 1. Additional figures and tables. [file 12951_2019_457_MOESM1_ESM.pdf]

Supporting Materials for

Specific drug delivery efficiently induced human breast tumor regression using a novel lipoplex by non-covalent association with anti-tumor antibodies

Yu-Ling Lin [1 †], Nu-Man Tsai [2,3], Chia-Hung Chen [4 †], Yen-Ku Liu [5], Chung-Jen Lee [6], Yi-Lin Chan[7], Yu-Shan Wang[8], Yuan-Ching Chang[9] Chi-Hsin Lin[10], Tse-Hung Huang[11,12,13], Chao Ching Wang[11], Kwan-Hwa Chi[8,*] and Kuang-Wen Liao[4,5,14,*]

* Author for correspondence:

Kuang-Wen Liao, Ph.D.

Department of Biological Science and Technology

National Chiao Tung University

No.75 Po-Ai Street, Hsinchu 30068, Taiwan

Tel: 886-3-5712121 ext 56955; Fax: 886-3-5729288

E-mail: liaonms@pchome.com.tw

Kwan-Hwa Chi, M.D.

Department of Radiation Therapy and Oncology

Shin Kong Wu Ho-Su Memorial Hospital

No.95, Wenchang Rd., Shilin Dist., Taipei City 11101, Taiwan

Tel: 886-2-28332211 ext 2273; Fax: 886-2-28377582

E-mail: M006565@ms.skh.org.tw

Targeting ability of drug/LPPC/Herceptin complexes *in vitro*

MCF7 cells ($5x10^5$ per each well) were coated in a 96-well microtiter plate overnight, and the plate was blocked with 1% skim milk for 1 h. The immunocomplexes such as Cur/LPPC/Herceptin or Cur/LPPC/Retuximab were applied to the MCF7 cell-coated plate and incubated for 1 h on ice. Both immunocomplexes contains 40 μg LPPC and 8.7 mM of curcumin. After washing the unbound immunocomplexes with PBST (0.05% Tween 20 in PBS), the cell-bound immunocomplexes were dissolved by DMSO, and the curcumin concentration was measured using an ELISA reader at 450 nm.

The pharmacokinetics of curcumin *in vivo*

Balb/c mice were intravenously injected with PBS, curcumin, curcumin/LPPC or curcumin/LPPC/Herceptin, and the blood samples were harvested from the tail vein at 0.5, 1, 8, 24, and 36 h. After centrifugation (1,100 × g, 4°C, 25 min), plasma samples were obtained and extracted with a 2-fold volume of ethyl acetate. The ethyl acetate extracts were evaporated, dissolved in 100 μl of DMSO, and assessed using an ELISA reader at 450 nm. All pharmacokinetic analyses were performed using the WinNonlin Standard Edition Version 5.3 (Scientific Consulting Inc., Apex, NC, USA). A non-compartmental model was utilized for both data fitting and parameter

estimation.

The bio-distribution of curcumin and tissue damage *in vivo*

NOD-SCID mice received subcutaneous injections of SKBR3 cells (1×10^7 cells in 100 μl of PBS). After the injection period, the animals were sacrificed. The tumors and organs were harvested, fixed in 10% buffered formalin saline and then embedded in paraffin blocks. Tissue sections (4-μm thickness) were prepared and stained with hematoxylin and eosin (H&E). The tissue morphology was examined using a microscope with 400X magnification. The fluorescence of curcumin and doxorubicin in the pathological sections was photographed under the fluorescence microscopy.

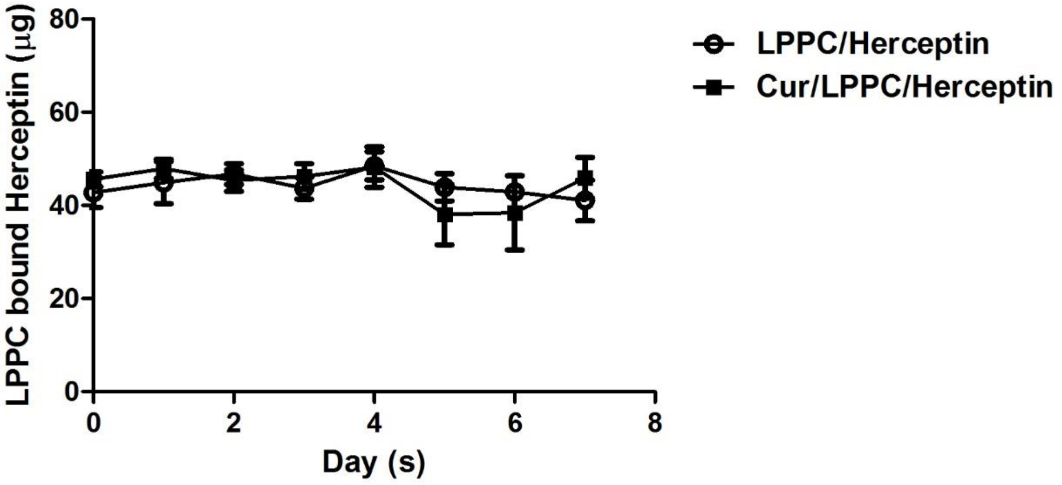

Figure S1. Stabilities of LPPC/Herceptin or Cur/LPPC/Herceptin. The amounts of LPPC bound Herceptin were measured by the Bradford assay. Representative data are shown as the mean ± SD of two independent experiments (n=4).

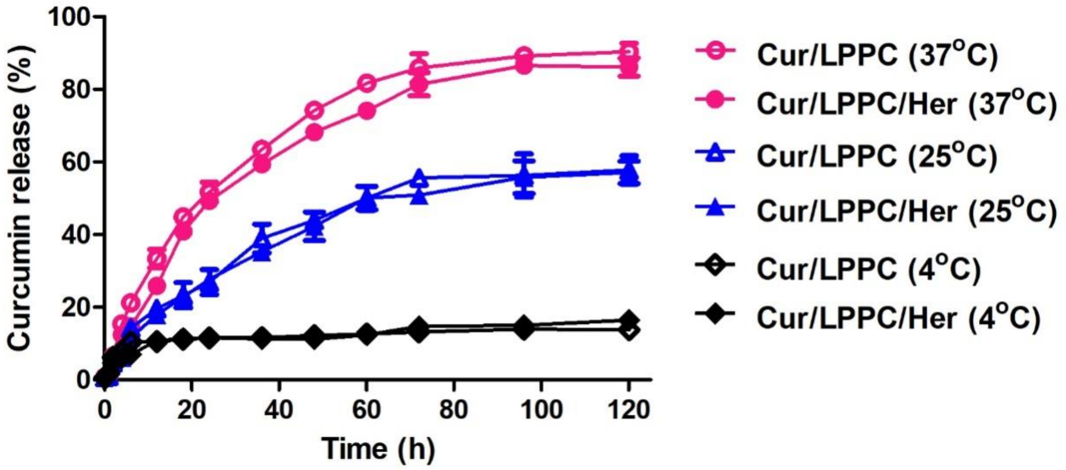

Figure S2. The effect of Herceptin association on curcumin release from the curcumin/LPPC complexes. Curcumin/LPPC or Curcumin/LPPC/Herceptin complexes were incubated in serum (10% FBS in PBS) at 0, 25 or 37°C. The concentration of curcumin in each supernatant was measured at various incubation time points and compared with the total curcumin concentration. All values represent the mean ± SD (n=3).

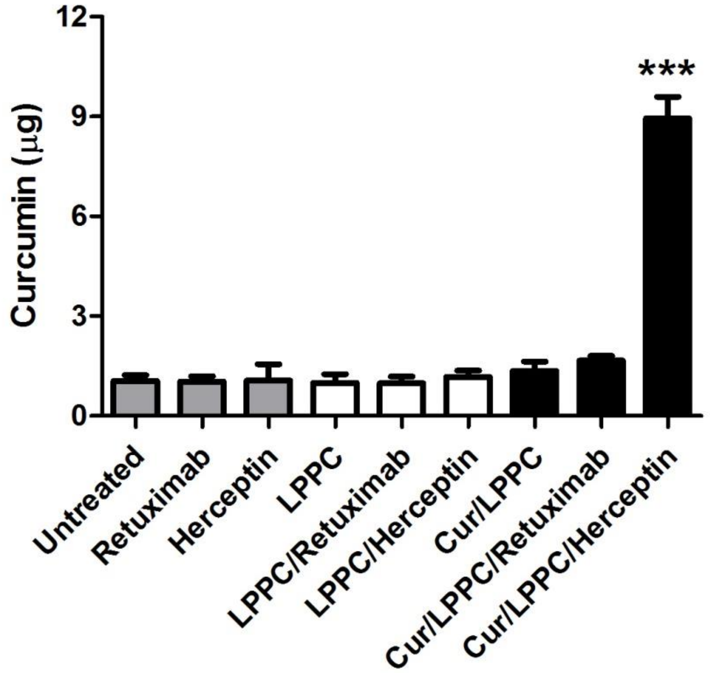

Figure S3. The effect of curcumin loading on the cell targeting of Cur/LPPC/Herceptin *in vitro*. MCF-7 cells were treated with Cur/LPPC/Herceptin for 1 h on ice. LPPC/Herceptin, LPPC/Retuximab, Cur/LPPC or Cur/LPPC/Retuximab were used as control. All immunocomplexes contained 40 μg LPPC and 8.7 mM of curcumin. The cell-bound Cur/LPPC/Herceptin were dissolved by DMSO and then the concentrations of curcumin were analyzed. All values represent the mean ± SD, ***p<0.001 (n=4).

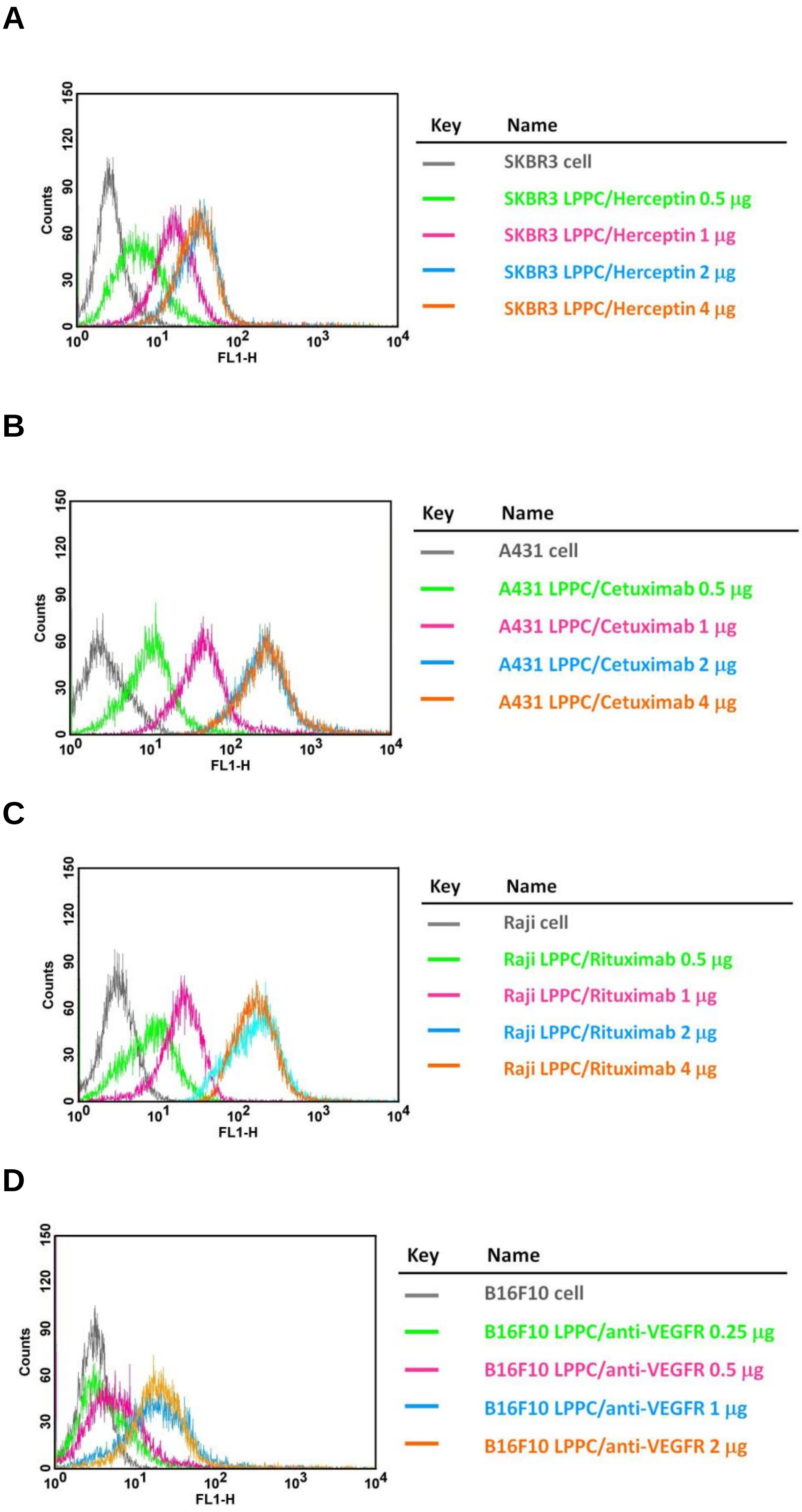
A
Counts
FL1-H
Key
Name
SKBR3 cell
SKBR3 LPPC/Herceptin 0.5 μg
SKBR3 LPPC/Herceptin 1 μg
SKBR3 LPPC/Herceptin 2 μg
SKBR3 LPPC/Herceptin 4 μg
B
Counts
FL1-H
Key
Name
A431 cell
A431 LPPC/Cetuximab 0.5 μg
A431 LPPC/Cetuximab 1 μg
A431 LPPC/Cetuximab 2 μg
A431 LPPC/Cetuximab 4 μg
C
Counts
FL1-H
Key
Name
Raji cell
Raji LPPC/Rituximab 0.5 μg
Raji LPPC/Rituximab 1 μg
Raji LPPC/Rituximab 2 μg
Raji LPPC/Rituximab 4 μg
D
Counts
FL1-H
Key
Name
B16F10 cell
B16F10 LPPC/anti-VEGFR 0.25 μg
B16F10 LPPC/anti-VEGFR 0.5 μg
B16F10 LPPC/anti-VEGFR 1 μg
B16F10 LPPC/anti-VEGFR 2 μg

E

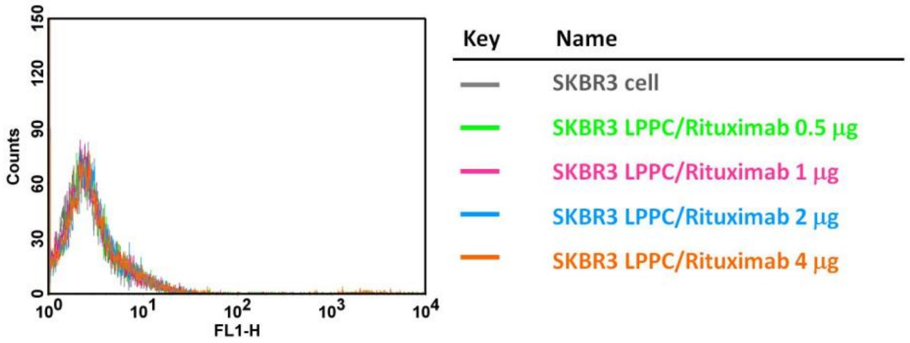

Figure S4. Cell targeting of DiO-labeled LPPC/antibody complexes. (A) SKBR3 (HER2 positive), (B) A431 (EGFR positive), (C) Raji (CD20 positive) and (D) B16 melanoma(VEGF positive)cells werestained with the DiO-labeled LPPC complexwith various antibodies at different doses. DiO-LPPC/Herceptin complex targeted the SKBR3 cells; DiO-LPPC/Cetuximab complex targeted the A431 cells; DiO-LPPC/Rituximab complex targeted the Raji cells; DiO-LPPC/anti-VEGFR1 antibodycomplex targeted theB16F10 melanoma cells. (E) DiO-LPPC-Rituximab complex did not target the SKBR3 cells.

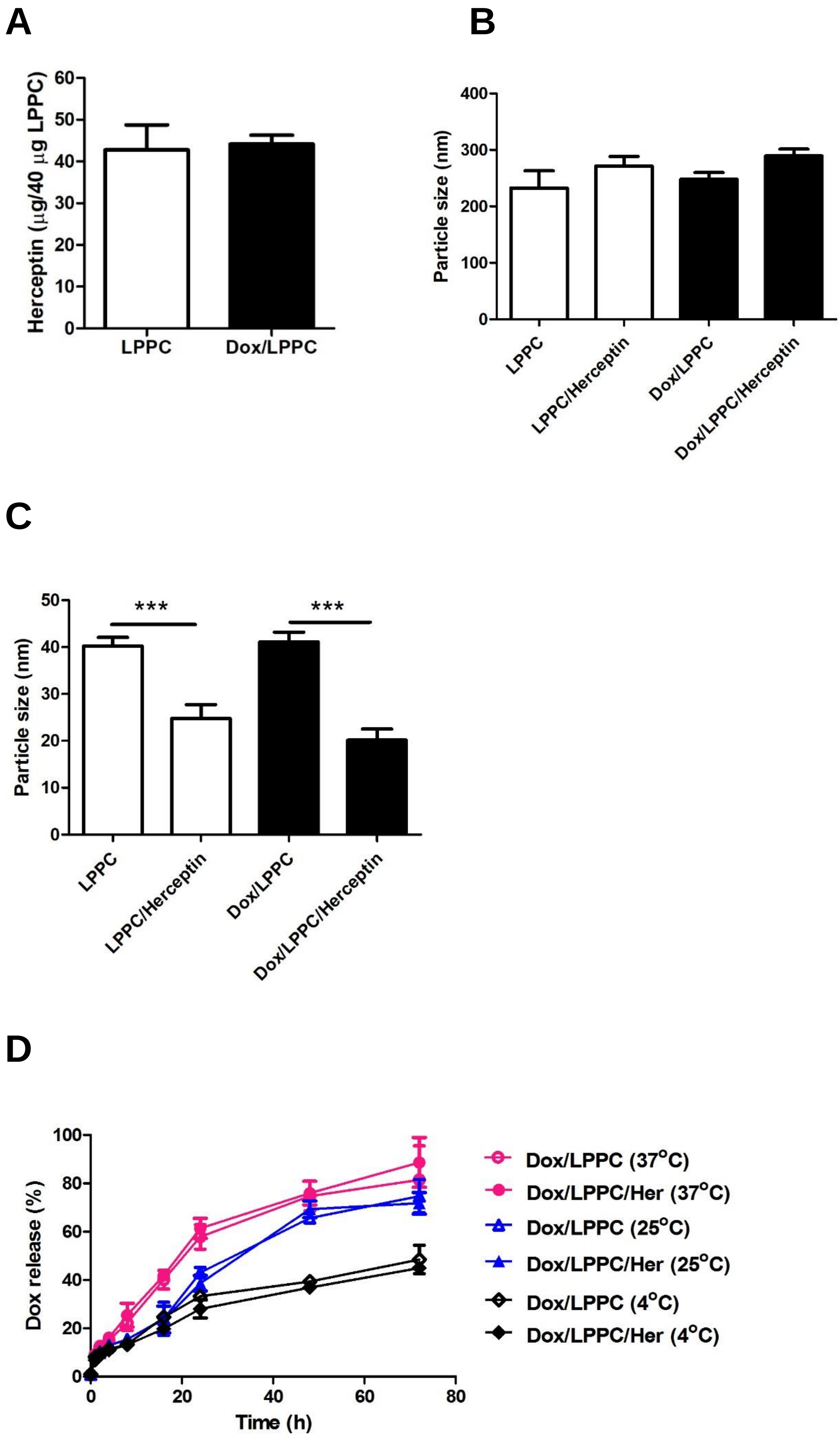
A
Herceptin (μg/40 μg LPPC)
60
50
40
30
20
10
0
LPPC
Dox/LPPC
B
Particle size (nm)
400
300
200
100
0
LPPC
LPPC/Herceptin
Dox/LPPC
Dox/LPPC/Herceptin
C
Particle size (nm)
50
40
30
20
10
0
***
***
LPPC
LPPC/Herceptin
Dox/LPPC
Dox/LPPC/Herceptin
D
Dox release (%)
100
80
60
40
20
0
0
20
40
60
80
Time (h)
Dox/LPPC (37°C)
Dox/LPPC/Her (37°C)
Dox/LPPC (25°C)
Dox/LPPC/Her (25°C)
Dox/LPPC (4°C)
Dox/LPPC/Her (4°C)

Figure S5. The characteristics of the Dox/LPPC/Herceptin complex. (A) The maximal-binding capacity of Herceptin to Dox/LPPC. Dox/LPPC (40 μg) was incubated with different amounts of Herceptin, and the maximal amount of bound protein was analyzed with the Bradford Assay. (B) The particle size and (C) zeta-potential of the Dox/LPPC/Herceptin complex. All values represent the mean ± SD (n=4). (D) Dox release from the Dox/LPPC complexes. Dox/LPPC or Dox/LPPC/Herceptin complexes were incubated in serum (10% FBS in PBS) at 0, 25 or 37°C. The concentration of Dox in each supernatant was measured at various incubation time points and compared with the total Dox concentration. All values represent the mean ± SD (n=3).

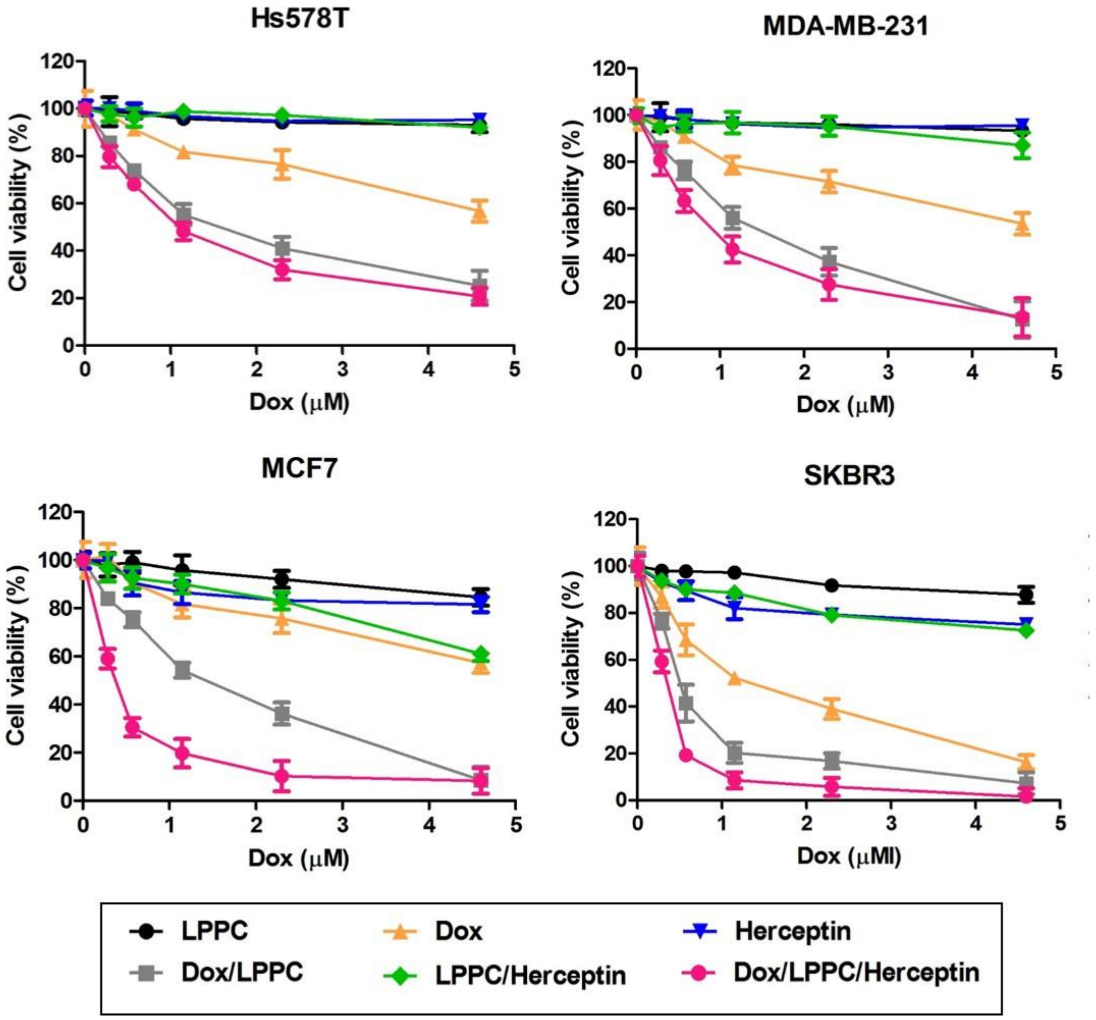

Figure S6. Cytotoxic effects of Dox/LPPC/Herceptin on HER2-negative or HER2-positive cell lines. Hs578T (HER2-), MDA-MB-231 (HER2-/+), MCF7 (HER2 +), and SKBR-3 (HER2++) cells were treated with 0 to 4.6μM Dox for the treatment of Dox/LPPC/Herceptin for 48 h. The dosages of LPPC, Herceptin, and LPPC/Herceptin were the same as those used for the Dox/LPPC/Herceptin treatments. Cell viability was assessed by MTT assay (n=4).

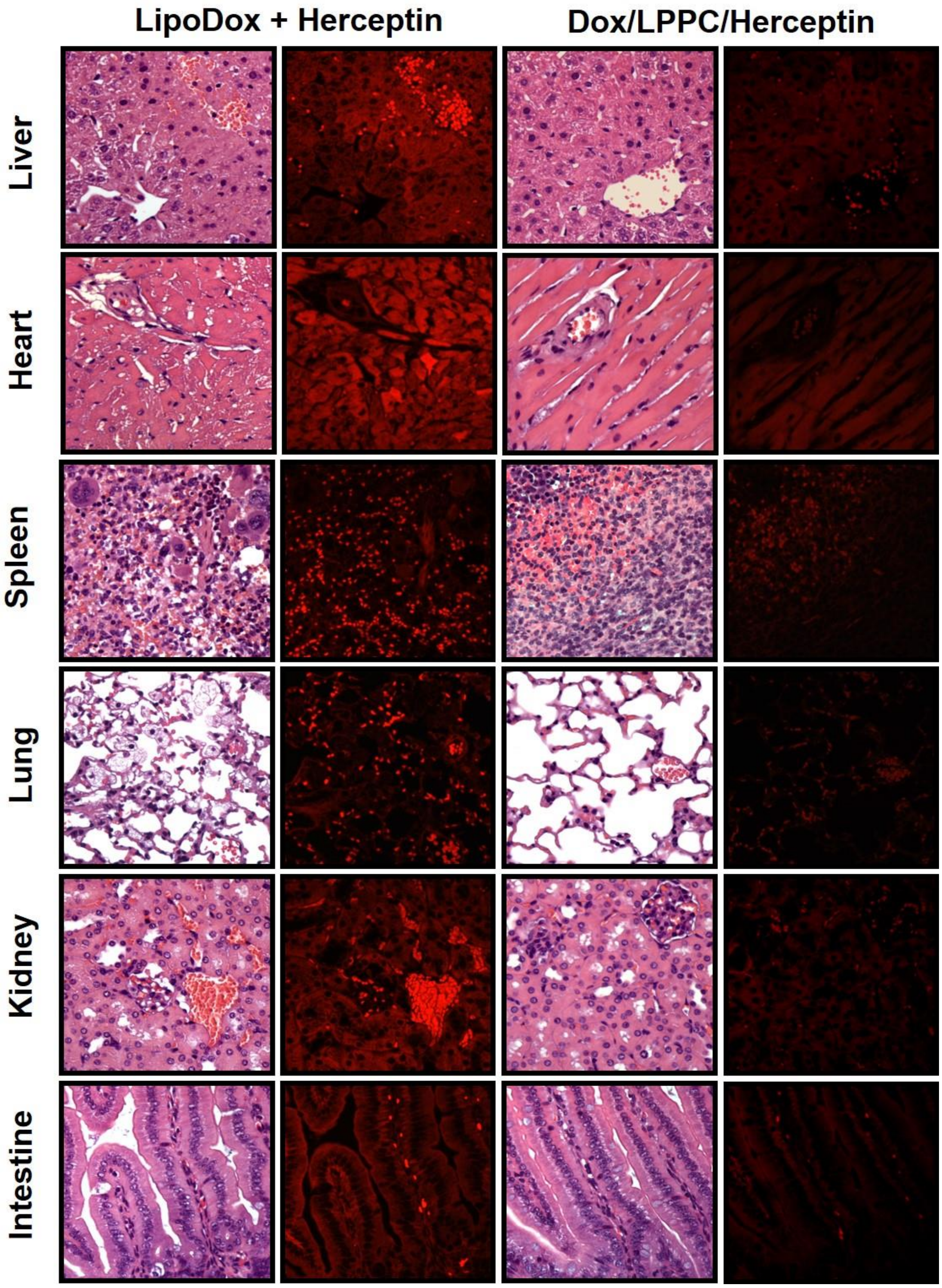

Figure S7. The bio-distribution of doxorubicin in normal tissue. NOD-SCID mice bearing SKBR3 tumors were injected intravenously with Lipodox plus either Herceptin or the Dox/LPPC/Herceptin complex at a dose of 5 mg doxorubicin/kg. The

mice were sacrificed at day 3. The histological sections of the organ samples were collected after sequential injections of Lipodox with either Herceptin or the Dox/LPPC/Herceptin complex. Dox accumulation was imaged using a fluorescent microscope; the red fluorescence indicates the Dox.

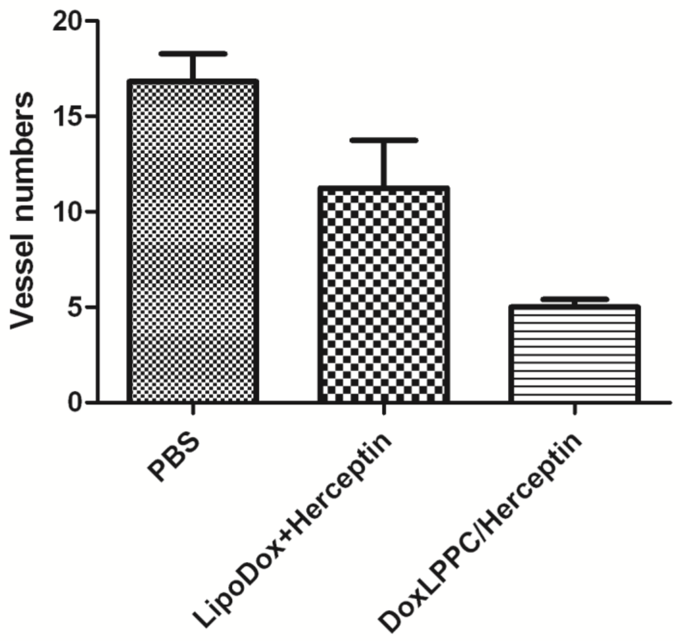

Figure S8. The anti-angiogenetic activity of Dox/LPPC/Herceptin.SKBR3 cells were implanted s.c. into NOD-SCID mice. Lipodox with Herceptin or the Dox/LPPC/Herceptin complex were injected via i.v. at a dose of 5 mg doxorubicin/kg. After a 72-h treatment period, the vessel numbers in tumors were calculated. The data represent the means ± SD (n=8).

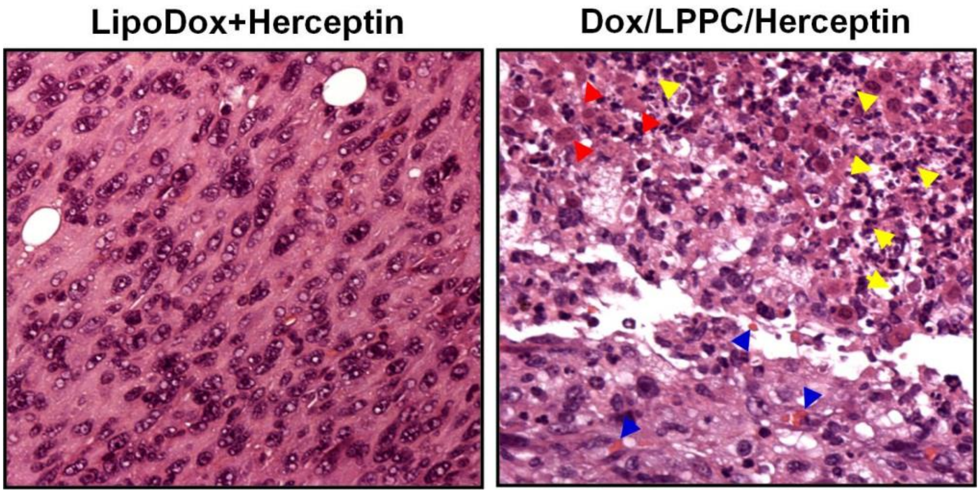

Figure S9. The levels of apoptosis and necrosis in Dox/LPPC/Herceptin-treated tumors. NOD-SCID mice bearing SKBR3 tumors were injected intravenously with Lipodox plus either Herceptin or the Dox/LPPC/Herceptin complex at a dose of 5 mg doxorubicin/kg. The mice were sacrificed at day 2. The histological sections of the tumor samples were collected after injection of the Dox/LPPC/Herceptin complex. Red arrows:cell necrosis; yellowarrows: apoptosis; blue arrows: hemorrhage.

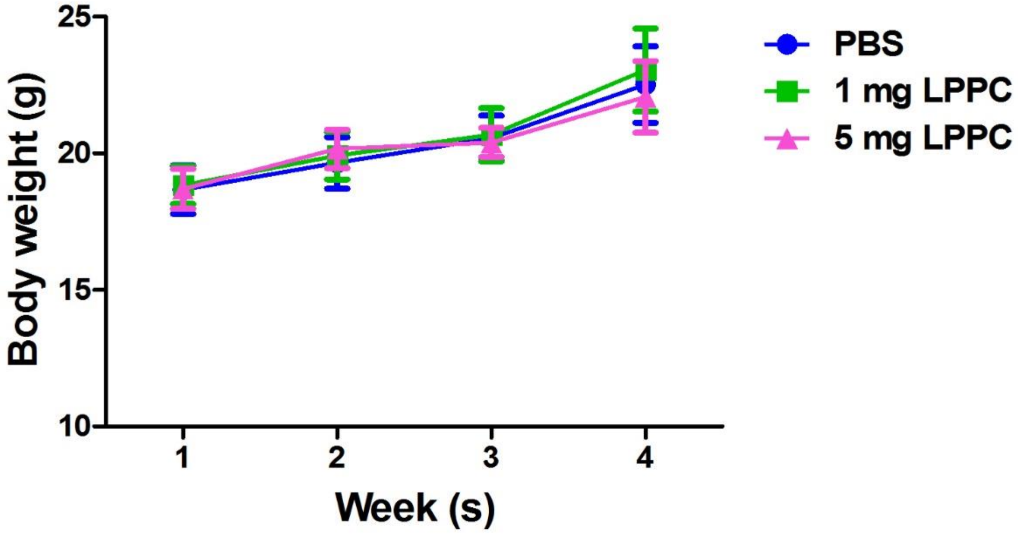

Figure S10. Average body weight of mice during treatment. BALB/c mice were treated with 5 mg LPPC per mouse for 4 weeks. The body weight of LPPC-treated mice were measured weekly. All values represent the mean ± SD (n=6).

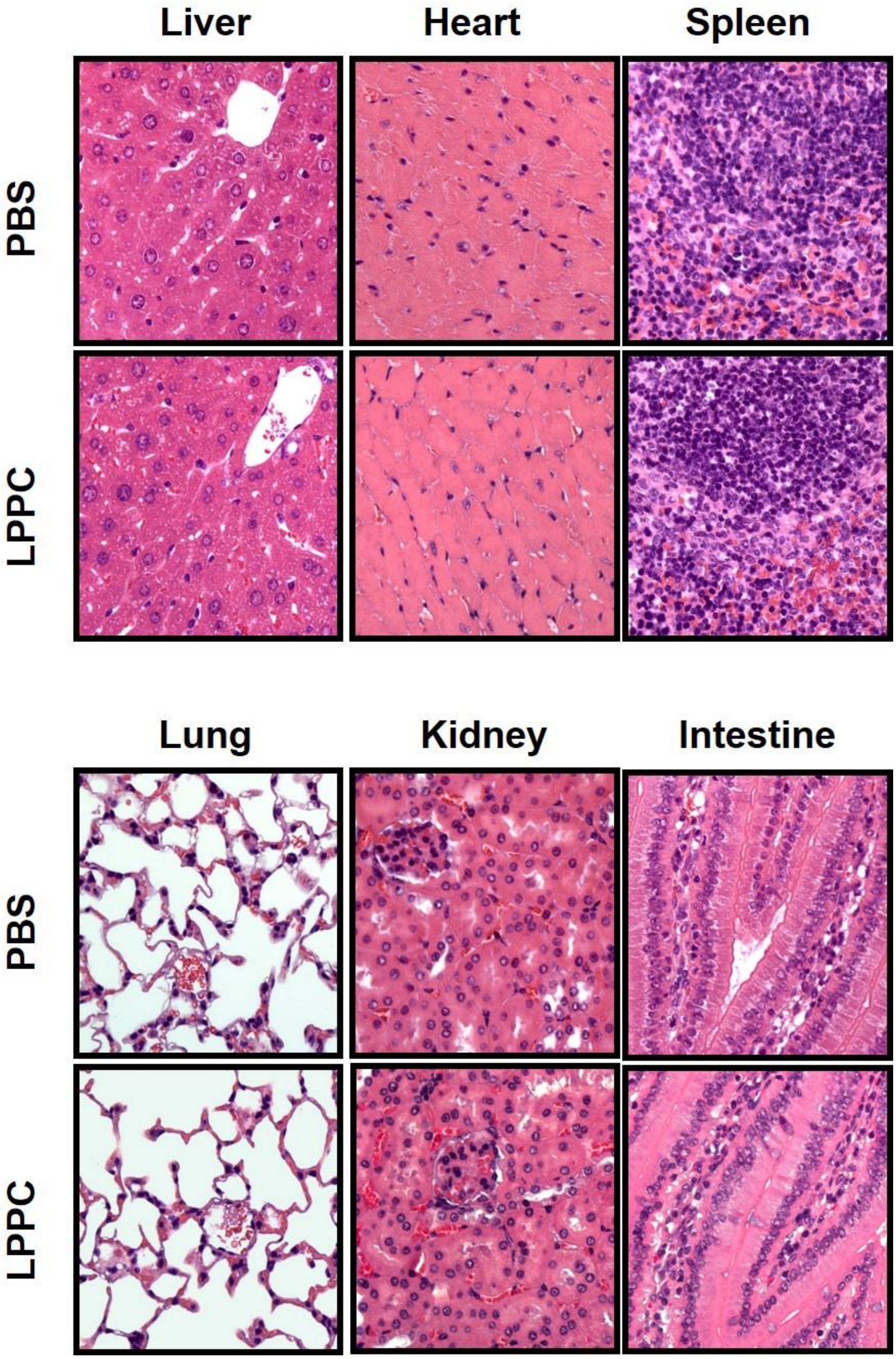

Figure S11. LPPC did not induce tissue damage in normal tissues. The BALB/cmice were treated with 5 mg LPPC per mouse for 4 weeks (n=6). The organs were

collected and evaluated by H&E staining (400X).

Table S1. The particle sizes and zeta-potentials of curcumin/LPPC/Herceptin.

Groups	Particle size (nm)	PDI	Zeta-potential (mV)
LPPC	232.5 ± 30.8	0.19 ± 0.04	40.2 ± 1.8
LPPC/Herceptin	271.6 ± 17.0	0.19 ± 0.12	24.8 ± 3.0
Cur/LPPC	249.6 ± 25.3	0.23 ± 0.09	40.2 ± 2.0
Cur/LPPC/Herceptin	278.7 ± 22.8	0.17 ± 0.12	24.5 ± 1.1
Dox/LPPC	248.1 ± 12.2	0.42 ± 0.05	41.1 ± 2.1
Dox/LPPC/Herceptin	289.4 ± 12.3	0.37 ± 0.13	20.2 ± 2.4

* The data represent the means ± SD (n=4).

Table S2. Effects of curcumin/LPPC/Herceptin on proliferation in different cell lines.

Cells	IC50 (μM)		
	Curcumin	Cur/LPPC	Cur/LPPC/Herceptin
Hs578T	12.46 ± 1.65	0.87 ± 0.07	0.59 ± 0.06
MDA-MB-231	12.15 ± 5.23	1.23 ± 0.12	0.57 ± 0.03
MCF7	10.21 ± 1.13	0.96 ± 0.23	0.27 ± 0.09
SKBR3	15.73 ± 3.70	1.28 ± 0.14	0.23 ± 0.01

* All values are mean ± SD of three independent experiment (n=6).

Table S3. Effects of Dox/LPPC/Herceptin on proliferation in different cell lines.

Cells	IC50 (μM)		
	Dox	Dox/LPPC	Dox/LPPC/Herceptin
Hs578T	6.55 ±1.22	1.58 ± 0.37	1.14 ± 0.15
MDA-MB-231	5.58 ± 1.02	1.54 ± 0.30	0.95 ± 0.12
MCF7	6.02 ± 0.71	1.44 ± 0.22	0.38 ± 0.04
SKBR3	1.32 ± 0.14	0.51 ± 0.06	0.35 ± 0.02

* All values are mean ± SD of two independent experiment (n=4).

Table S4. The serum albumin (ALB), total protein (TP) and glucose (GLU) levels of LPPC-treated mice.

Biochemical factors	Treated period (weeks)	Control (PBS)	PEG_{1500}/ LPPC	
			1 mg	5 mg
ALB (g/dL)	1	3.65 ± 0.12	3.56 ± 0.05	3.64 ± 0.04
	2	3.50 ±0.54	3.51 ± 0.25	3.49 ± 0.37
	3	3.50 ± 0.27	3.54 ± 0.21	3.51 ± 0.23
	4	3.56 ± 0.26	3.83 ± 0.28	3.56 ± 0.26
TP (g/dL)	1	5.43 ± 0.17	5.53 ± 0.02	5.62 ± 0.16
	2	5.39 ± 0.24	5.26 ± 0.14	5.63 ± 0.10
	3	5.61 ± 0.29	5.37 ± 0.21	5.11 ± 0.14
	4	5.35 ± 0.44	5.76 ± 0.53	5.33 ± 0.45
GLU (mg/dL)	1	180.2 ± 35.3	156.3 ± 12.0	157.6 ± 25 5
	2	124.3 ± 14.4	117.1 ± 14.6	120.2 ± 14.4
	3	192.5 ± 26.3	173.9 ± 27.8	151.7 ± 14.4
	4	172.7 ± 56.0	222.3 ± 41.3	176.5 ± 54.0

* All values represent the mean ± SD (n=6).

Table S5. The serum alanine aminotransferase (ALT), aspartate aminotransferase (AST), creatine kinase (CK) and lactate dehydrogenase (LDH) levels of LPPC-treated mice.

Biochemical factors	Treated period (weeks)	Control (PBS)	PEG_{1500}/LPPC	
			1 mg	5 mg
ALT (IU/L)	1	58.0 ± 4.1	58.3 ± 4.2	60.0 ± 1.6
	2	56.2 ± 1.3	56.5 ± 9.4	55.2 ± 7.1
	3	60.7 ± 7.0	57.4 ± 3.7	58.8 ± 9.0
	4	59.1 ± 9.4	58.3 ± 8.3	57.5 ± 6.7
AST (IU/L)	1	135.9 ± 60.6	126.1 ± 17.2	139.3 ± 60.6
	2	136.6 ± 35.8	137.4 ± 35.3	134.7 ± 40.3
	3	137.0 ± 45.4	137.2 ± 45.2	138.8 ± 34.8
	4	137.2 ± 29.6	136.7 ± 22.4	138.5 ± 35.1
CK (IU/L)	1	476.3 ± 121.2	474.4 ± 101.4	446.5 ± 141.8
	2	444.3 ± 244.4	479.6 ± 122.3	485.3 ± 288.2
	3	459.4 ± 156.3	462.0 ± 132.2	472.1 ± 276.7
	4	468.0 ± 139.7	469.8 ± 181.4	477.8 ± 188.7
LDH (IU/dL)	1	386.0 ± 40.5	385.5 ± 12.3	381.7 ± 22.2
	2	388.0 ± 13.2	379.0 ± 28.6	383.1 ± 41.9
	3	371.1 ± 45.3	380.8 ± 50.1	372.9 ± 40.8
	4	385.3 ± 36.2	383.2 ± 30.4	383.8 ± 38.5

* All values represent the mean ± SD (n=6).

Table S6. The creatinine, urea and lactose levels of LPPC- treated mice.

Biochemical factors	Treated period (weeks)	Control (PBS)	PEG_{1500}/LPPC	
			1 mg	5 mg
CRE (mg/dL)	1	0.18 ± 0.01	0.18 ± 0.01	0.18 ± 0.02
	2	0.26 ± 0.05	0.23 ± 0.09	0.26 ± 0.09
	3	0.19 ± 0.03	0.20 ± 0.01	0.16 ± 0.03
	4	0.18 ± 0.06	0.21 ± 0.06	0.19 ± 0.02
Urea (mg/dL)	1	22.0 ± 4.0	19.7 ± 2.6	20.7 ± 1.2
	2	25.6 ± 0.8	19.4 ± 1.1	23.0 ± 1.8
	3	35.3 ± 4.2	27.1 ± 1.6	22.0 ± 3.1
	4	29.8 ± 6.1	31.9 ± 3.9	27.7 ± 1.1
Lactate (mg/dL)	1	109.1 ± 15.9	112.2 ± 16.8	121.8 ± 17.0
	2	104.0 ± 71.6	105.1 ± 56.4	99.9 ± 62.2
	3	96.9 ± 53.5	89.9 ± 44.3	90.9 ± 3.8
	4	117.2 ± 34.8	112.2 ± 25.0	89.8 ± 20.5

* All values represent the mean ± SD (n=6).

Table S7. Pharmacokinetic parameters of curcumin in mice after i.v. injection with curcumin, curcumin/LPPC or curcumin/LPPC/Herceptin.

Pharmacokinetic parameters	Curcumin†	Cur/LPPC	Cur/LPPC/Herceptin
Cmax (μg/mL)	15.5 ± 0.6	15.9 ± 0.7	16.7 ± 1.4
AUC (minμg/mL)	115.4 ± 22.9	307.7 ± 23.2*	343.2 ± 18.4*
t1/2 (h)	< 1	13.1 ± 2.3	13.8 ± 2.7
Cl (mL/min/kg)	353.7 ± 70.3	130.5 ± 10.0*	116.7 ± 6.1*
MRT (min)	0.49 ± 0.4	18.4 ± 3.2*	19.0 ± 4.4*
Vss (mL/kg)	3549.5 ± 1322.2	2458.8 ± 358.3	2318.0 ± 395.9

Data are expressed as mean ± SD, * $p < 0.01$, significantly different from the curcumin group.

† The concentrations of curcumin in all groups are 40 mg/kg.

Cmax: maximum concentration; AUC: area concentration versus time curve; t1/2: elimination half-life; Cl: clearance; MRT: mean resident time; Vss: steady-state volume of distribution.
